# Isoquinoline Alkaloid Contents in *Macleaya cordata* Extracts and Their Acetylcholinesterase and Butyrylcholinesterase Inhibition

**DOI:** 10.3390/molecules27113606

**Published:** 2022-06-03

**Authors:** Tomasz Tuzimski, Anna Petruczynik, Małgorzata Szultka-Młyńska, Mateusz Sugajski, Bogusław Buszewski

**Affiliations:** 1 Department of Physical Chemistry, Medical University of Lublin, Chodźki 4a, 20-093 Lublin, Poland; 2 Department of Inorganic Chemistry, Medical University of Lublin, Chodźki 4a, 20-093 Lublin, Poland; 3 Department of Environmental Chemistry and Bioanalytics, Faculty of Chemistry, Nicolaus Copernicus University, Gagarina 7, 87-100 Torun, Poland; szultka.malgorzata@wp.pl (M.S.-M.); mateusz.sugajski@o2.pl (M.S.); bbusz@chem.umk.pl (B.B.); 4 Centre for Modern Interdisciplinary Technologies, Nicolaus Copernicus University in Torun, Wileńska 4, 87-100 Torun, Poland; 5 Kujawsko-Pomorskie Heritage Center and Kujawsko-Pomorskie Science and Technology Center Prof. Jana Czochralskiego, Czerwona Droga 8, 87-100 Torun, Poland

**Keywords:** *Macleaya cordata*, isoquinoline alkaloids, cholinesterase activity inhibition, HPLC-DAD, LC-MS/MS

## Abstract

An important strategy for treating neurodegenerative disorders is to maintain the levels of acetylcholine in the synaptic cleft by blocking the cholinesterases. Searching for new effective compounds with inhibited acetylcholinesterase and butyrylcholinesterase activity is one of the most significant challenges of the modern scientific research. The aim of this study was the optimization of the condition for cholinesterase activity determination by high-performance liquid chromatography coupled with diode array detector (HPLC-DAD) in terms of concentrations of enzymatic reaction mixture components, temperature of incubation, and incubation time. In vitro investigation of acetylcholinesterase and butyrylcholinesterase activity inhibition by some isoquinoline alkaloids and extracts obtained from the aerial part and roots of *Macleaya cordata* collected in May, July, and September. Acetylcholinesterase and butyrylcholinesterase activity inhibition of the extracts obtained from the plant had not been tested previously. The application of the HPLC method allowed eliminating absorption of interfering components, for example, alkaloids such as sanguinarine and berberine. The HPLC method was successfully applied for the evaluation of the acetylcholinesterase inhibitory activity in samples such as plant extracts, especially those containing colored components adsorbing at the same wavelength as the adsorption wavelength of 5-thio-2-nitro-benzoic acid, which is the product of the reaction between thiocholine (product of the hydrolysis of acetyl/butyrylthiocholine reaction) with Ellman’s reagent. Moreover, liquid chromatography coupled with a triple quadrupole mass spectrometer (LC–QqQ–ESI–MS/MS) analysis allowed evaluating the identification of relevant bioactive compounds in the obtained plant extracts. The investigated alkaloids, especially sanguinarine and chelerythrine, and all the *Macleaya cordata* extracts, especially the extract obtained from the aerial part collected in May, exhibited very high cholinesterase activity inhibition. HPLC-DAD was also applied for the kinetics study of the most active alkaloids sanguinarine and chelerythrine. Our investigations demonstrated that these plant extracts can be recommended for further in vivo experiments to confirm their cholinesterase inhibition activity.

## 1. Introduction

Neurodegenerative disorders are a growing public health problem due the rising incidence and low effectiveness of current treatments [1,2]. Alzheimer’s disease is one of the most common age-related irreversible and progressive neurodegenerative disorders characterized by dementia, memory loss, a decline in language skills, and cognitive impairment with aging.

Several hypotheses of neurodegenerative diseases, including cholinergic, amyloid, τ-protein, calcium dyshomeostasis, and isoprenoid change, have been proposed. An important strategy for treating neurodegenerative diseases is to maintain the levels of acetylcholine in the synaptic cleft by blocking the activity of cholinesterases [3]. Cholinesterases play an important role in the regulation of neurotransmission by catalyzing the degradation of neurotransmitter acetylcholine [4]. Currently, the cholinergic hypothesis associates the memory impairment and cognitive disturbances in Alzheimer’s disease with the large loss of cholinergic hippocampal neurons as a response to acetylcholine deficit which causes a cholinergic hypofunction and deficits of different cortical cholinergic markers. Under normal conditions, acetylcholine is dominantly hydrolyzed by acetylcholinesterase [5]. However, when the level of acetylcholinesterase declines, butyrylcholinesterase can play a function compensation role for acetylcholinesterase to some extent to maintain normal cholinergic pathways. Currently, there is no effective therapy for the most common neurodegenerative disease, Alzheimer’s disease, and three of the most prescribed drugs (donepezil, rivastigmine, and galantamine) for the treatment are cholinesterase inhibitors. However, these drugs only treat symptoms and are inefficient for advanced stages of the disease. Therefore, it is very important to search for new and more effective drugs against neurodegenerative diseases.

Different plant extracts containing alkaloids have been tested as cholinesterase inhibitors, for example, indole alkaloids from *Psychotria nemorosa* [6], piperidine alkaloids from *Senna spectabilis* [7], sesquiterpene alkaloids from *Huperzia quadrifariata* [8], diterpenoid alkaloids from *Aconitum hemsleyanum* [9] and from *Aconitum laeve* [10], β-carboline alkaloids [11]. Most often, alkaloids belonging to the group of Amaryllidaceae alkaloids have been tested as cholinesterase inhibitors, e.g., alkaloids from *Narcissus pseudonarcissus* [12], *Clinanthus microstephium* [13], Amarylidaceae alkaloids, namely gigantelline, gigantellinine, and gigancrinine from *Crinum jagus* [14], *Crinum jagus*, *Clivia miniata, Phaedranassa lehmannii*, *Eucharis bonplandii*, *Eucharis caucana*, and *Hippeastrum elegans* [15,16], *Zephyranthes carinata* [15], *Zephyranthes fosteri* [17], *Crinum*, *Habranthus* and *Zephyranthes* species [18], *Pancratium parvum* [19], Genus *Lycoris* [20], *Rhodolirum speciosum* [21], *Narcissus pseudonarcissus* [22].

For the determination of alkaloid contents in plant materials, various analytical techniques are applied. Most often, alkaloids are determined by chromatographic methods such as liquid chromatography coupled with tandem mass spectrometry (LC-MS/MS) [3,7,9,23], ultra-high-performance liquid chromatography with diode array detection (UPLC-DAD) [4], gas chromatography with mass spectrometry (GC-MS) [8,13,15,24].

Many plants containing alkaloids have been tested as cholinesterase inhibitors. Often, for in vitro anticholinesterase activity determination, assays are based on Ellman’s method with various modifications.

For example, Plazas et al. tested 41 alkaloid extracts from nine *Zanthoxylum* species for their acetylcholinesterase and butyrylcholinesterase inhibition activity [25]. Nine isoquinoline alkaloids obtained from the *Corydalis mucronifera* extract were investigated as acetylcholinesterase inhibitors [26]. Isoquinoline alkaloids from three *Cryptocarya* species were also determined as cholinesterase inhibitors [27]. Using Ellman’s method, the alkaloid extract of *Fumaria officinalis* [28], alkaloid fractions of *Berberis aetnensis* and *Berberis libanotica* [24], the most active alkaloid obtained from *Illigera grandiflora* [29], bisbenzylisoquinoline alkaloids cyclanoline, obamegine, homoaromoline, and nor-N′-chondrocurine obtained from roots of *Cissampelos pareira* [30], a select alkaloid from *Cryptocarya densiflora* were also tested as acetylcholinesterase inhibitors [31].

A fishing platform using immobilized capillary enzyme reactors in combination with liquid chromatography–mass spectrometry was applied for determination of acetylcholinesterase inhibition activity of an extract from *Mahonia bealei* [32].

Currently, chromatography is increasingly used for determination of cholinesterase activity inhibition. Wang et al. also used HPLC for testing the acetylcholinesterase inhibitory activity of anthocyanins. These compounds present markedly diverse colors at different values of pH, which makes it difficult to determine the absorbance in the color-rendering experiment [33]. Online acetylcholinesterase activity inhibition determination by high-performance liquid chromatography–mass spectrometry (LC-MS) together with an immobilized enzyme reactor has also been rarely applied [34]. Ultrafiltration liquid chromatography–mass spectrometry (UFLC-MS) is also an efficient method that can be applied to rapidly screen and identify ligands binding to acetylcholinesterase [35].

In the present study, for the determination of acetylcholinesterase inhibition activity, the HPLC-DAD method was applied, which allows a much more accurate determination of this activity compared to the commonly used spectrophotometric method. The use of chromatography to determine the inhibition of acetylcholinesterase activity allowed for the elimination of the measurement of adsorption at the same wavelength by Ellman’s reaction product and other components, e.g., alkaloids such as sanguinarine, chelerythrine. The chromatographic method is suitable for plant extracts with deep color. Moreover, based on the obtained fragmentation patterns with the use of LC-QqQ-ESI-MS/MS, the studied alkaloids were exactly identified in plant extracts. The kinetics study for the most active alkaloids chelerythrine and sanguinarine was also performed. The aim of this work was also to evaluate in vitro acetylcholinesterase and butyrylcholinesterase inhibitory activities of extracts obtained from *Macleaya cordata* roots and aboveground parts collected at three harvesting times during one growing season.

## 2. Results and Discussion

### 2.1. Determination of Alkaloid Contents in Plant Extracts

Alkaloid standards were chromatographed using a Polar RP column in an eluent system containing acetonitrile, water, and 0.04 M 1-butyl-3-methylimidazolium tetrafluoroborate in a gradient system described in section “Experimental”. The chromatographic condition was based on the previously published procedure applied for determination of isoquinoline alkaloids after an appropriate modification [36,37]. The LOQ values for investigated alkaloids were determined in a previous study and were 0.0123 mg/mL for chelerythrine, 0.0288 mg/mL for protopine, and 0.0371 mg/mL for sanguinarine [37].

**Figure 1:**
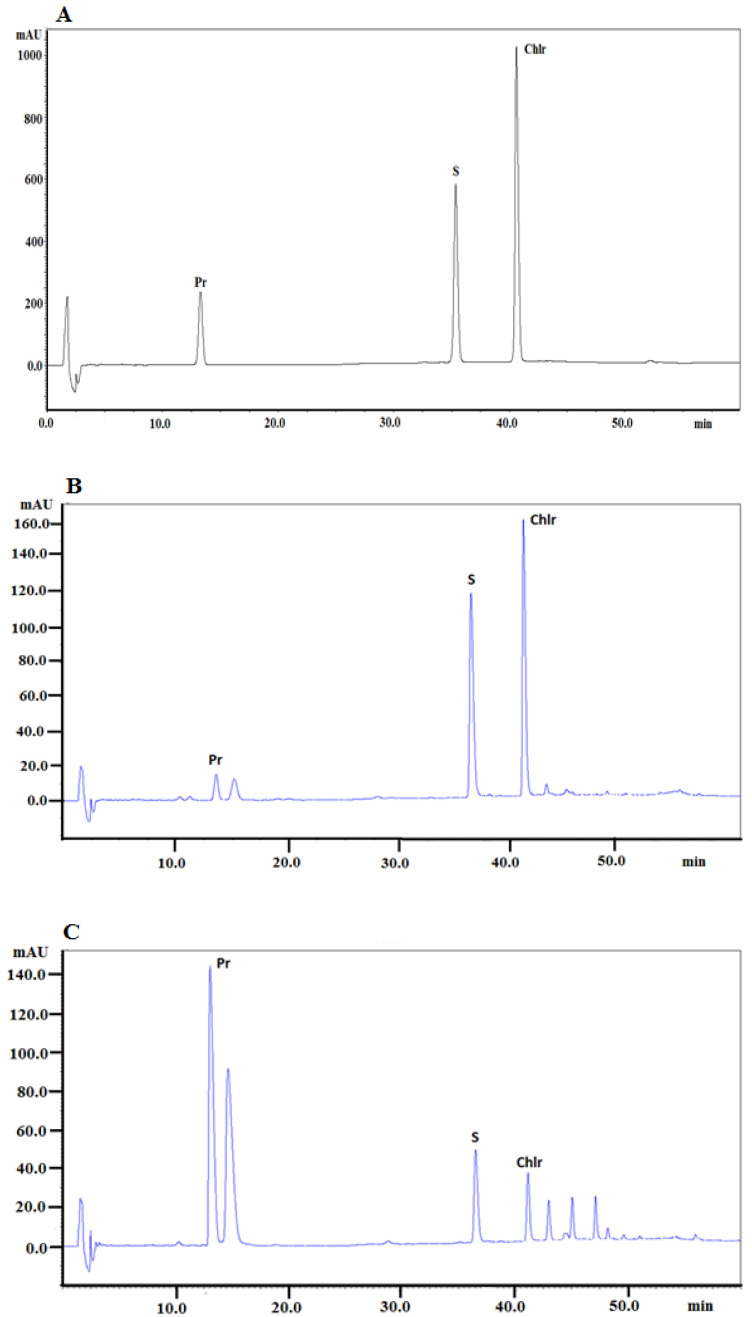
(**A**) HPLC-DAD chromatogram obtained for a mixture of alkaloid standards, (**B**) HPLC-DAD chromatogram obtained for the extract from the *Macleaya cordata* aerial part collected in May, (**C**) HPLC-DAD chromatogram obtained for the extract from the *Macleaya cordata* root collected in May. Chlr: chelerythrine; Pr: protopine; S: Ssnguinarine. The acquisition wavelength was set at 240 nm.

The same chromatographic system was applied to determine alkaloids in plant extracts obtained from *Macleaya cordata* roots and aboveground parts collected in May, July, and September. The presence of alkaloids in the investigated extracts was identified by comparison of retention times with the retention times of alkaloid standards, UV–Vis spectra, and additionally confirmed by MS spectra. The chromatogram obtained for the investigated alkaloid standards is presented in Figure 1A. Figure 1B presents a typical chromatogram obtained for the extract from the *Macleaya cordata* aerial part collected in May and Figure 1C presents a typical chromatogram obtained for the extract from *Macleaya cordata* root collected at the same time.

Significant differences were found in the average content of alkaloids in the extracts obtained both from the aerial part or roots and in the extracts obtained from the plant material collected at various vegetation steps (Table 1). The higher content of protopine was determined in the extracts obtained from roots compared to the extracts from the aerial part. For example, in the root extracts collected in May, 16.576 mg of protopine per g of dry plant material were determined, while in the extracts from the aerial part collected at the same time, only 1.361 mg of the alkaloid per g of dry plant material was determined. Sanguinarine and chelerythrine were determined in higher contents in the extracts obtained from the aerial part compared to the extracts from roots, e.g., in the extract from the aerial part collected in May, 4.091 and 5.395 mg/g of dry plant material of sanguinarine and chelerythrine were found, respectively, while in the extract from roots also collected in May, 1.647 and 1.105 mg/g of dry plant material of sanguinarine and chelerythrine were determined, respectively.

Great differences in alkaloid contents were also observed in the extracts obtained from the plant material collected in various months. The highest content of protopine was found in the extract from roots collected in May before the flowering of the plant (16.576 mg/g of dry plant material). The content of protopine in roots significantly decreased in the extracts obtained from roots collected in July, during the flowering of the plant (7.658 mg/g of dry plant material), and was similar in plant material to the sample collected in September (8.693 mg/g of dry plant material). The highest content of protopine in the aerial part was observed in the plant material collected in July (2.288 mg/g of dry plant material) and was similar to the plant material collected in May and September (about 1.3 mg/g of dry plant material).

The highest content of sanguinarine was determined in the aerial part collected in May (4.091 mg/g of dry plant material); then, the content of the alkaloid decreased in the plant material collected in July and September (2.888 and 2.413 mg/g of dry plant material, respectively). In the roots collected in May, 1.647 mg of sanguinarine per g of dry plant material was determined; then, a slight increase in the content of this alkaloid was observed in the material collected in July and a decrease in the content in the material collected in September.

Chelerythrine was found in the highest concentration in the extract obtained from the aerial part collected in May (5.395 mg/g of dry plant material). In the plant material collected in July and September, a significantly lower content of the alkaloid was determined (3.474 and 3.257 mg/g of dry plant material, respectively). In the extracts obtained from roots, the highest concentration of chelerythrine was determined when the plant material was collected in July (1.544 mg/g of dry plant material), the lowest—in the extract from the plant material collected in September (0.883 mg/g of dry plant material). Table 2 presented the content of investigated isoquinoline alkaloids in the dry residue of plant extracts.

### 2.2. LC-QqQ-ESI-MS/MS for the Identification of Selected Alkaloids in Plant Extracts

Tandem mass spectrometry with electrospray ionization (ESI-QqQ-MS/MS) was applied in the positive ionization mode in order to develop a sensitive method for the identification of the four studied alkaloids. First, the full scan mass spectra were recorded in order to select parent ions. Then, the fragmentation of each studied compounds was performed in the product’s ion mode. Collision energy (CE) was optimized in the range of 20–35 eV (the product’s ion spectra were recorded every 5 eV). Hence, the studied alkaloids were identified based on the accurate mass of quasimolecular ions, MS/MS spectra, and the fragmentation pathway.

The selected alkaloids were identified in the plant extracts obtained from various parts (aerial part and roots) of *Macleaya cordata* collected in May, July, and September. Full MS and MS/MS spectra of *m*/*z* 50–500 were acquired for each target compound. Moreover, because the ESI mass spectrum was obtained from an acidic solution which put the studied alkaloids into a positively charged quaternary nitrogen form, their peaks of highest mass corresponded to the actual molecular mass, [M + H]^+^.

Table S1 summarizes the chromatographic information and MS-based information (adduct forms, observed mass, product ions). Highly abundant protonated molecule [M+H]^+^ ions of all the studied alkaloids were observed in the ESI mass spectra due to the strong basicity of the secondary or tertiary amine group. Hence, relevant isoquinoline alkaloids were identified based on MS spectra for allocryptopine (*m*/*z* = 370.15), chelerythrine (*m*/*z* = 348.20), protopine (*m*/*z* = 354.30), and sanguinarine (*m*/*z* = 332.10) respectively. Moreover, the presence of them in real plant samples regarding different morphological parts (aerial part and root) was confirmed by MS/MS spectra, collision induced dissociation (CID) of the peak of the most intense ion.

The identification of the studied alkaloids in the plant extracts was based on the accurate mass measurements, the isotopic distribution of parent ions, and the study of their fragmentation patterns. Subsequently, the parent ions fragmented in different characteristic main ways.

Alpha allocryptopine gave the parent ion at *m*/*z* = 370.15. Fragments at *m*/*z* 206.05 and *m*/*z* = 165.10 were generated by the retro Diels–Adler (RDA) reaction. The ion at *m*/*z* = 206.05 further fragmented generating fragment ions at *m*/*z* = 189.10 (the loss of the –OH group) and *m*/*z* = 188.05 (loss of H_2_O). The mentioned ions were the most dominant signals in the MS/MS spectrum of α-allocryptopine, indicating that the loss of H_2_O or –OH following the RDA reaction could be the major fragmentation pathway. Additionally, the parent ion of α-allocryptopine might undergo α-cleavage, producing the ions at *m*/*z* = 181.10. Moreover, the parent ion might break up into ions at *m*/*z* 352.70 (C_21_H_22_NO_4_^+^), 290.15 (C_19_H_14_O_3_^+^), and 275.10 (C_18_H_11_O_3_^+^) after the loss of H_2_O, -OCH_3,_ and -CH_3_. The signals at *m*/*z* = 336.30 and *m*/*z* = 306.20 might be produced by the loss of CH_4_ from the ion at *m*/*z* = 352.70 and by the loss of -CH_3_ from the ion at *m/z* = 321.10, respectively. In case of protopine, fragment ions at *m/z* = 205.70 and 146.90 in the MS/MS spectrum are generated by the RDA (retro Diels–Adler reaction) C ring opening, but given the presence of hydroxyl groups, the product ions at m/*z* = 334.10 and *m*/*z*= 189.05 are probably formed by loss of H_2_O from the molecular ion and from the *m/z* = 205.70 ion. In case of sanguinarine, the MS/MS spectrum exhibited fragment ions at *m*/*z* = 316.60 [M–CH_3_]^+^, *m*/*z* = 304.20 [M–CO]^+^ and at *m*/*z* = 274.20 [M – (CH_2_O + CO)]^+^. Based on the obtained results, similar fragmentation could be assumed for chelerythrine at *m*/*z* = 348.20, which was mainly fragmented to *m*/*z* = 303.95 and *m*/*z* = 274.60.

**Figure 2:**
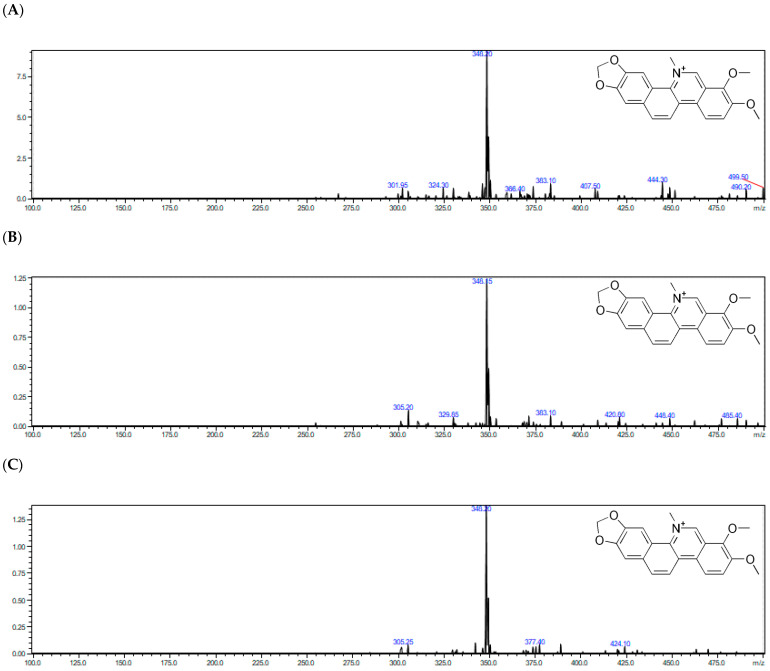

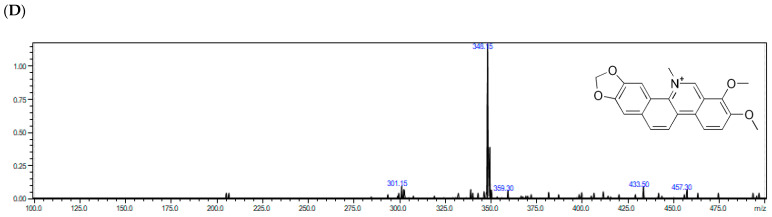
Representative full-scan MS spectra obtained with the use of ESI–Q-TOF–MS/MS for chelerythrine from the *Macleaya cordata*’s extracts: (**A**) aerial part collected in May, (**B**) root collected in May, (**C**) aerial part collected in July, (**D**) root collected in July, as well as representative product ion MS/MS spectra obtained with the use of ESI-Q-TOF-MS/MS for chelerythrine (**E**), protopine (**f**), and sanguinarine (**G**) from the *Macleaya cordata* extracts.

The representative full-scan MS and product ion MS/MS spectra obtained with the use of ESI–Q-TOF–MS/MS for chelerythrine from the *Macleaya cordata* extracts are shown in Figure 2.

### 2.3. Optimization of Cholinesterase Activity Determination

Optimization of acetylcholinesterase, butyrylcholinesterase, acetylthiocholine iodide, butyrylthiocholine iodide, and 5′-dithiobis-(2-nitrobenzoic) acid concentrations, time, and temperature of reaction mixture activation were performed before investigations of alkaloid standards and plant extracts in terms of anticholinesterase activity.

**Figure 3:**
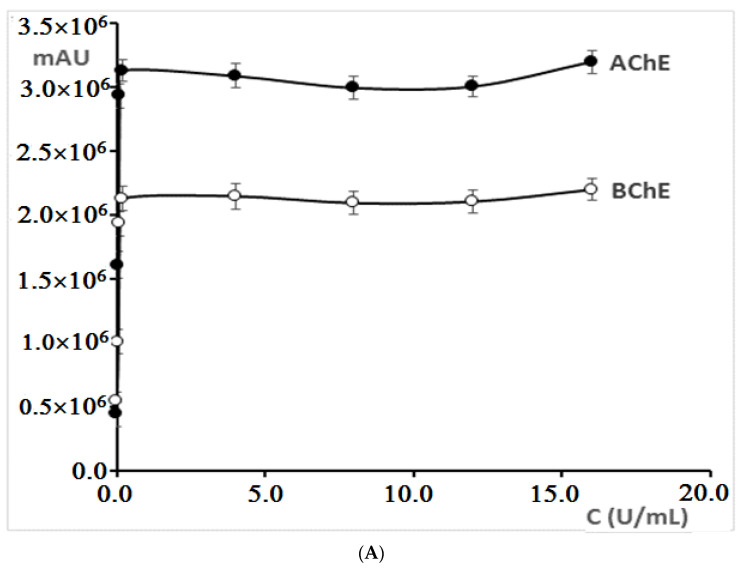

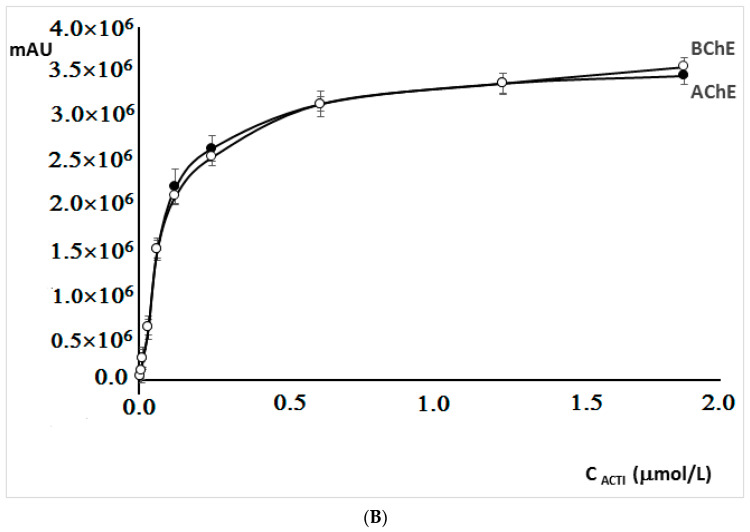

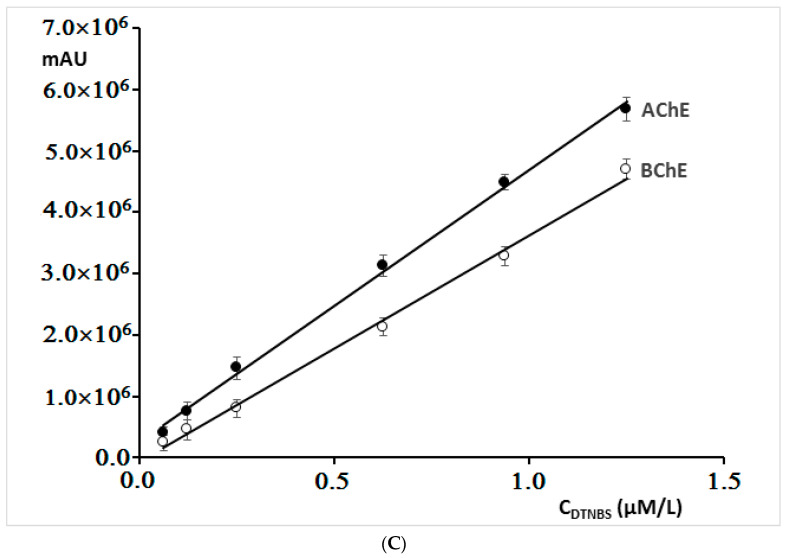
(**A**) Dependence of concentration of cholinesterases on the peak area. Error bars represent standard errors. (**B**) Dependence of ACTI and BCTI concentrations in the reaction with cholinesterases on the peak area. Error bars represent standard errors. (**C**) Dependence of the DTNB concentration in reaction with cholinesterases on the peak area. Error bars represent standard errors. (**D**) Dependence of the temperature of incubation in reaction with cholinesterases on the peak area. Error bars represent standard errors. (**E**) Dependence of the incubation time in reaction with acetylcholinesterases on the peak area. Error bars represent standard errors.

For reactions with both acetylcholinesterase and butyrylcholinesterase, an increase in the peak area for 5-thio-2-nitro-benzoic acid (TNBA), which is the product of reaction between thiocholine (product of the hydrolysis of acetyl/butyrylthiocholine reaction) and Ellman’s reagent, was obtained in the concentration range from 0.01 U/mL to 0.2 U/mL (Figure 3A). With a concentration higher than 0.2 U/mL, the peak area practically did not change. Therefore, in further investigations, cholinesterases at the concentration of 0.2 U/mL were used.

The increase of acetylthiocholine iodide and butyrylthiocholine iodide concentrations from 0.003125 µM to 0.625 µM resulted in a significant increase in the 5-thio-2-nitro-benzoic acid peak area, while the further increase in iodides concentrations did not significantly affect the area of the peak (Figure 3B). For this reason, the concentration of 0.625 µM was chosen for further experiments.

By increasing the concentration of 5′-dithiobis-(2-nitrobenzoic acid) in mixtures containing both cholinesterases, a linear dependence of the peak area on the concentration of this reagent was obtained (Figure 3C). In further investigations, concentration of 0.167 µM was applied, because already at this concentration, a sufficiently large peak area was obtained.

The influence of the reaction mixture incubation temperature on 5-thio-2-nitro-benzoic acid peak area was also examined. The mixture was incubated in the range of temperatures from 20 °C to 55 °C. In both cases, initially, a significant increase in the peak area was observed (from 20 °C to 30 °C) (Figure 3D). The maximum of the peak area was observed at about 37 °C which corresponds to the temperature of the human body. Further, the incubation temperature increase reduced the area of the peaks obtained for the enzymatic reaction product. Therefore, in further experiments, the samples were activated at 37 °C.

Increasing the incubation time initially resulted in an increase in the 5-thio-2-nitro-benzoic acid peak area (from 0 to about 15 min), and then its decrease was observed (Figure 3E). A greater reduction in the peak area with a longer incubation time was observed, especially for the reaction mixture containing acetylcholinesterase.

### 2.4. Determination of Cholinesterase Inhibition Activity of Alkaloid Standards

The in vitro cholinesterase inhibition activity of alkaloid standards chelerythrine, protopine, and sanguinarine was performed using the HPLC-DAD method. The chromatographic method for determination of this activity is much more accurate compared to the spectrophotometric method, especially for compounds such as sanguinarine and chelerythrine, which show very high adsorption at the wavelength of λ = 412 nm at which the determination of 5-thio-2-nitro-benzoic acid is usually performed. The dose-dependent isoquinoline alkaloid response curves of the cholinesterase inhibitory activity are presented in Figure S1.

The IC_50_ values were calculated for each alkaloid using concentrations ranging from 0.1 to 200 µM. In this study, galantamine and rivastigmine were used as the positive control. The IC_50_ values of acetylcholinesterase and butyrylcholinesterase inhibition by isoquinoline alkaloid standards are given in Table 3. All the investigated alkaloids showed inhibitory activity against both enzymes. The highest acetylcholinesterase and butyrylcholinesterase inhibition activity was determined for chelerythrine (IC_50_ = 0.72 and 2.15, respectively). IC_50_ = 1.028 μM and 3.554 μM obtained by modified Ellman’s method for chelerythrine against acetylcholinesterase and butyrylcholinesterase, respectively, were previously reported [25]. All the investigated alkaloids exhibited higher IC_50_ values against butyrylcholinesterase compared to the IC_50_ value against acetylcholinesterase. However, the most active alkaloid chelerythrine exhibited higher butyrylcholinesterase activity inhibition with the IC_50_ value of 2.15 µM compared to galantamine and rivastigmine with the IC_50_ values of 5.31 and 8.38 µM, respectively.

### 2.5. Determination of Cholinesterase Inhibition Activity of Plant Extracts

At the next step of experiments, cholinesterase inhibition activity of the extracts obtained from the *Macleaya cordata* roots and aerial part collected in May, July, and September was investigated. The curves of the cholinesterase activity inhibition response to the dose of plant extracts are presented in Figures S2 and S3.

The in vitro anticholinesterase activity was significantly different for the extracts obtained both from the aerial part and roots from the extracts obtained from plant material collected in various months (Table 4). This behavior may be a result of the presence of alkaloids in the investigated extracts. The higher cholinesterase inhibition activity was obtained for the extracts from the aerial part of *Macleaya cordata* compared to the extracts from roots. The highest activity against both enzymes was determined for the extract obtained from the plant material collected in May (IC_50_ = 9.61 and 21.17 μg/mL against acetylcholinesterase and butyrylcholinesterase, respectively). In the extracts, the highest content of the most anticholinesterase-active alkaloids chelerythrine and sanguinarine was also determined. The high activity against acetylcholinesterase was also found for the extracts from the aerial parts collected in July and September (IC_50_ = 17.42 and 12.94 μg/mL, respectively). These extracts also exhibited high butyrylcholinesterase inhibition activity with IC_50_ = 28.04 μg/mL for the extract obtained from the plant material collected in July and IC_50_ = 25.74 μg/mL for the extract from the aerial part collected in September. In these extracts, high contents of more active alkaloids were determined (more than 2 mg/g of plant material of sanguinarine and more than 3 mg/g of plant material of chelerythrine). The lowest in vitro activity against acetylcholinesterase and butyrylcholinesterase was observed for the extract from roots collected in September, with IC_50_ values 59.14 and 64.28 μg/mL, respectively. In the extract, the lowest content of chelerythrine (0.883 mg/g of plant material) and sanguinarine (1.233 mg/g of plant material) was also determined. Previous anticholinesterase studies testing various plant extracts containing isoquinoline alkaloids reported different activity with different IC_50_ values. For example, the extract obtained from *Fumaria officinalis* exhibited inhibitory activities against acetylcholinesterase and butyrylcholinesterase with IC_50_ values of 39.23 and 40.32 μg/mL, respectively [28]; the alkaloid fractions of *Berberis aetnensis* and *Berberis libanotica* exhibited acetylcholinesterase inhibition activity with IC_50_ of 24.5 and 82.4 μg/mL, respectively, and antibutyrylcholinesterase activity with IC_50_ of 114.5 and 56.2 μg/mL, respectively [24]. The results demonstrated that the extracts obtained from of *Macleaya cordata* showed high anticholinesterase activity compared to the previously reported activity of various extracts containing isoquinoline alkaloids.

**Figure 4:**
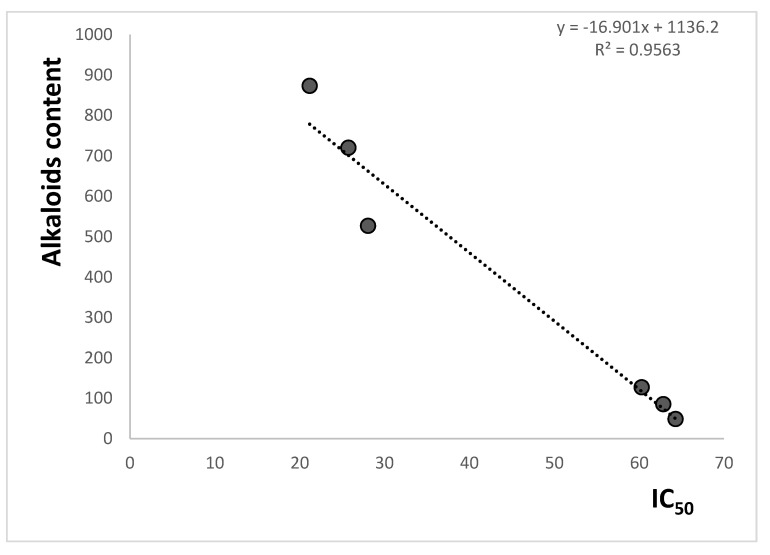
Correlation between the sum of chelerythrine and sanguinarine contents and butyrylcholinesterase activity inhibition as IC_50_ of the investigated plant extracts.

Correlations between the inhibition of cholinesterase activity expressed as IC_50_ values and the content of the investigated isoquinoline alkaloids and their sum were also examined (Figure 4 and Table 5). High values of the correlation coefficients were obtained for both the content of individual alkaloids and their total content. Especially, high correlations between activity and alkaloid contents were obtained for more active alkaloids chelerythrine: r = 0.9678 for correlation with antiacetylcholinesterase activity and r = 0.9749 for correlation with antibutyrylcholinesterase activity; sanguinarine: r = 0.9702 and 0.9815 for correlations with antiacetylcholinesterase and antibutyrylcholinesterase activities, respectively. The lower r values were obtained for less active protopine: 0.8540 and 0.8485 for correlations with antiacetylcholinesterase and antibutyrylcholinesterase activities, respectively. High r values were also obtained for the correlation between the sum of the content of chelerythrine and sanguinarine with cholinesterase activity (r = 0.9691 for the correlation with activity against acetylcholinesterase and r = 0.9779 for the correlation with activity against butyrylcholinesterase). The high r values obtained for correlation with chelerythrine and sanguinarine contents can indicate a very significant influence of these isoquinoline alkaloids on cholinesterase activity inhibition properties of the *Macleaya cordata* extracts. The investigated extracts also contain other alkaloids which may also influence their anticholinesterase properties.

Many reports have shown that low concentrations of isoquinoline alkaloids such as sanguinarine and chelerythrine have no observable toxic effects. For example, the most active compounds, chelerythrine and sanguinarine, exhibited low toxicity in swine or human cell lines [38,39]. Animals treated in the range of 5 mg/kg body weight did not display any results of hematological, biochemical, or histological assay different from controls [38]. A 32P-post-labeling assay also proved that no DNA adducts with sanguinarine and chelerythrine were detected in pig livers [38]. Based on biotransformation using rat liver microsomes of sanguinarine and chelerythrine, it was found that isolated metabolites were less toxic than the original compounds [39]. However, another report described a significant toxicity of these alkaloids [40].

Due to high anticholinesterase activity and often reported relatively low toxicity, further research on the composition, toxicity, and related anticholinesterase properties are advisable.

### 2.6. Kinetic Analysis of the Cholinesterase Inhibition

**Figure 5:**
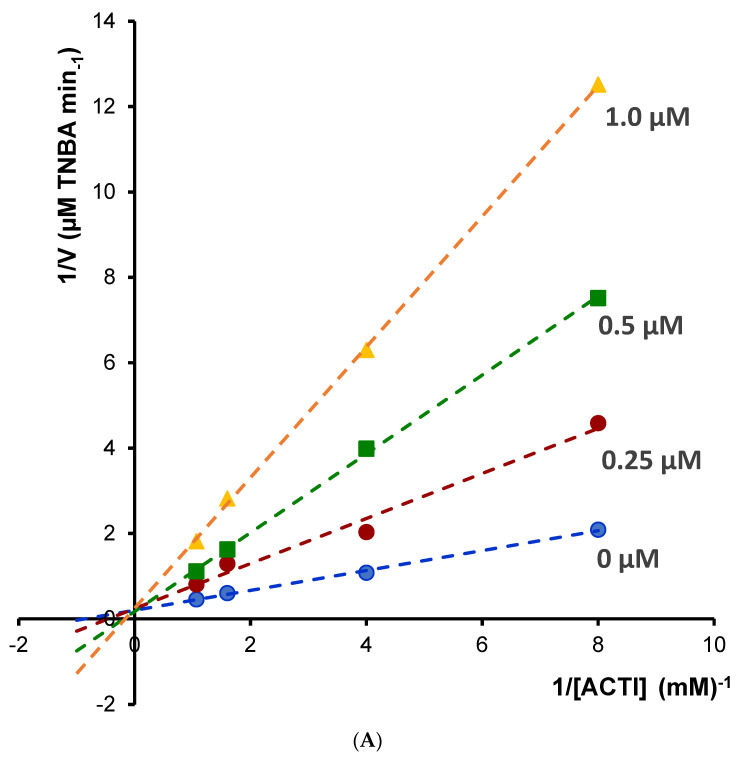

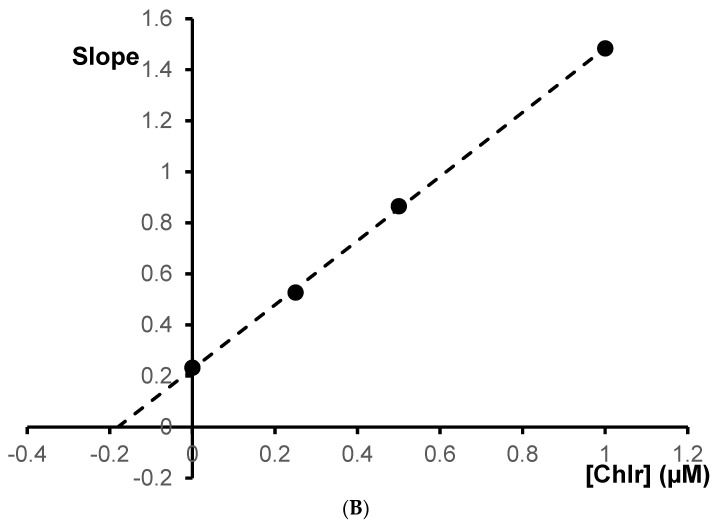
(**A**) Lineweaver–Bulk plot of acetylcholinesterase activity over a range of substrate concentration for chelerythrine, (**B**) determination of inhibition constants (K_i_) by plotting the slope of the primary Lineweaver–Burk plot vs. chelerythrine concentration.

**Figure 6:**
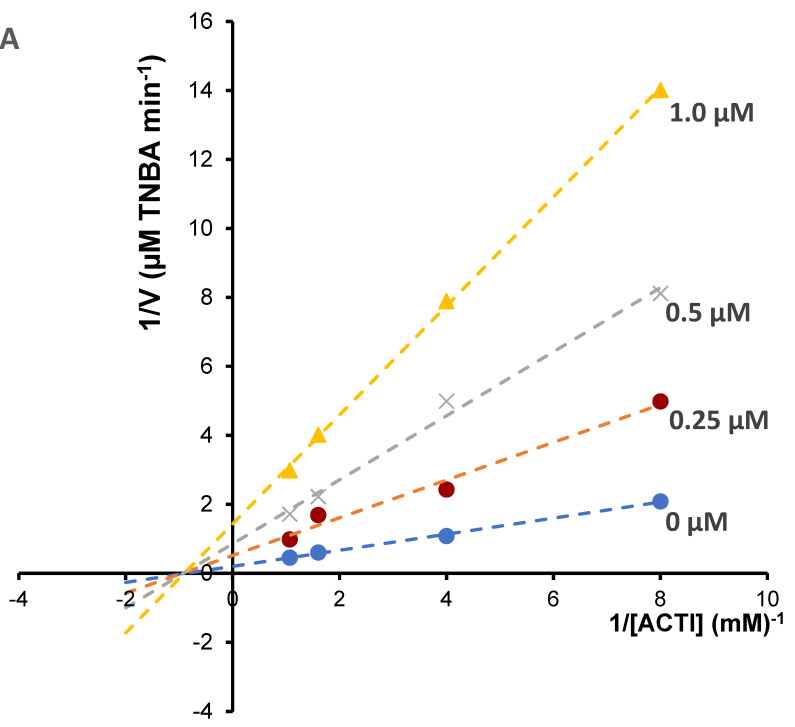

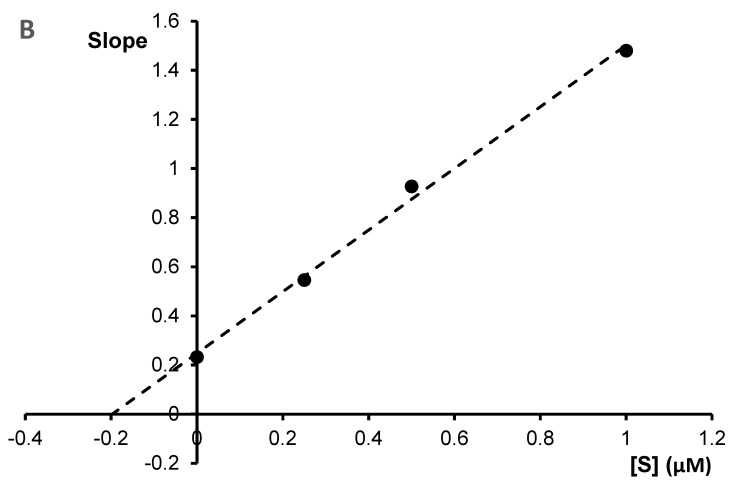
(**A**) Lineweaver–Bulk plot of acetylcholinesterase activity over a range of substrate concentrations for sanguinarine, (**B**) determination of inhibition constants (K_i_) by plotting the slope of the primary Lineweaver–Burk plot vs. sanguinarine concentration.

Enzyme kinetic study was performed for the most potent inhibitors of acetylcholinesterase; chelerythrine and sanguinarine. The type of inhibition and inhibition constants (Ki value) of the compounds were investigated using Lineweaver–Burk and Dixon plots (Figure 5 and Figure 6). The results showed that the acetylcholinesterase inhibition was competitive for chelerythrine (Figure 5A) and noncompetitive for sanguinarine (Figure 6A).

The Ki values were estimated by plotting the slope of the individual Lineweaver–Burk plots versus the inhibitor concentrations (Figure 5B and Figure 6B). For chelerythrine the estimated Ki was 0.18 µM, while for sanguinarine, the Ki was 0.20 µM.

### 2.7. Molecular Docking

#### 2.7.1. Docking Analysis for Acetylcholinesterase

Using the Molecular Operating Environment software, the best binding site for AChE was determined. Fifteen possible ligand binding zones were detected and the results are shown in the table. The first zone was used for further analysis because it had the highest score and size (Table 6).

**Figure 7:**
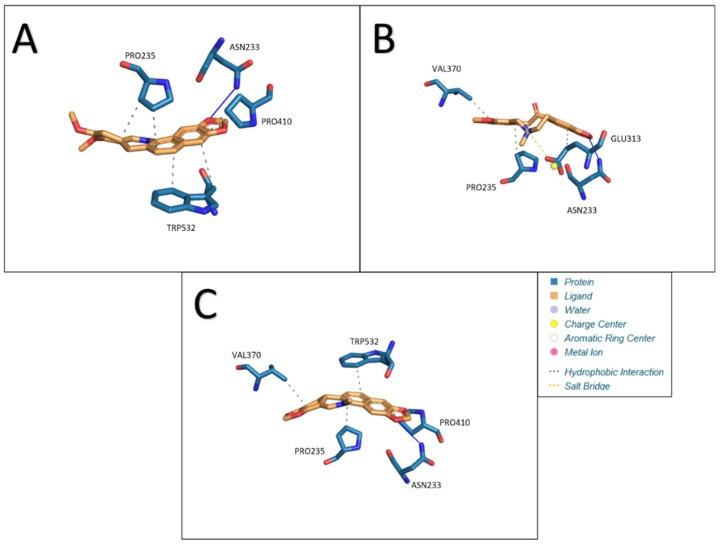
Interactions between AChE and ligands: (**A**) chelerythrine, (**B**) protopine, (**C**) sanguinarine.

Attachment of 3 ligands was performed in the selected zone: chelerythrine, protopine, sanguinarine. Docking of acetylcholinesterase with chelerythrine resulted in the formation of five hydrophobic interactions (PRO235, PRO235, PRO410, TRP532, and TRP532) and one hydrogen bond (ASN233). The lengths of the resulting bonds are as follows: 3.56, 3.57, 3.63, 3.24, 3.93, and 3.69. Under the given conditions, a structure with an Estimated Free Energy of Binding of −7.50 kcal/mol and an estimated inhibition constant of 3.17 μM was obtained. Interactions between acetylcholinesterase and ligands: A- chelerythrine, B- protopine, C- sanguinarine are presented in Figure 7.

Replacing the ligand with protopine allowed for three hydrophobic interactions (PRO235, GLU313, and VAL370), one hydrogen bond (ASN233), and the formation of one salt bridge (GLU313). The lengths of the resulting bonds are as follows: 3.44, 3.39, 3.51, 2.88 and 5.01. Estimated Free Energy of Binding −6.51 kcal/mol and estimated inhibition constant 16.99 μM.

The last ligand used for docking with AChE was sanguinarine. In this case, four hydrophobic bonds (PRO235, VAL370, PRO410, and TRP532) and one hydrogen bond (ASN233) were formed. The lengths of the bonds formed are: 3.68, 3.56, 3.70, 3.24 and 3.75. Estimated Free Energy of Binding was −7.50 kcal/mol and estimated inhibition constant 3.14 μM. The results obtained are presented in the Table 7.

#### 2.7.2. Docking Analysis for Butyrylcholinesterase

Fifteen potential zones for ligand docking were detected in the butyrylcholinesterase structure. These zones are shown in the Table 8. As with acetylcholinesterase, the zone with the highest score was selected. In the selected zone chelerythrine, protopine and sanguinarine were attached.

**Figure 8:**
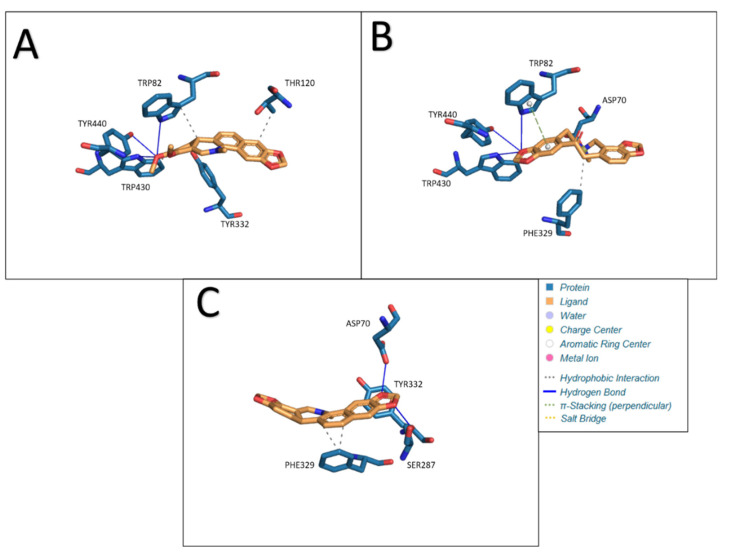
Interactions between BChE and ligands: (**A**) chelerythrine, (**B**) protopine, (**C**) sanguinarine.

Chelerythrine as a ligand formed four hydrophobic bonds (TRP82, THR120, TYR332, and TRP430) and three hydrogen bonds (TRP82, TRP430, and TYR440) in our study. The lengths of the resulting bonds are, respectively: 3.99, 3.40, 3.88, 3.49, 3.80, 3.06 and 3.30. The calculated Estimated Free Energy of Binding was −8.18 kcal/mol and the estimated inhibition constant was 1.01 μM. Interactions between butyrylcholinesterase and ligands: A- chelerythrine, B- protopine, C- Sanguinarine are presented in Figure 8.

The use of protopine as a ligand in docking with BChE resulted in the formation of one hydrophobic interaction (PHE329), three hydrogen bonds (TRP82, TRP430 and TYR440), one π-Stacking (perpendicular) bond (TRP82) and one salt bridge (ASP70). The lengths of the resulting bonds are as follows: 3.40, 4.02, 2.95, 3.68, 4.54, and 4.99. Under the given conditions, a structure with an Estimated Free Energy of Binding of −8.36 kcal/mol and an estimated inhibition constant of 0.75uM was obtained.

Sanguinarine docked to BChE produces three hydrophobic bonds (PHE329, PHE329, and TYR332) and two hydrogen bonds (ASP70 and SER287). The lengths of the resulting bonds are: 3.61, 3.67, 3.25, 3.11, and 2.63. Estimated Free Energy of Binding was −8.14 kcal/mol and an estimated inhibition constant of 1.06uM. The results obtained are shown in the Table 8.

## 3. Experimental

### 3.1. Apparatus and HPLC Conditions

Analysis was performed using an LC-20AD Shimadzu (Shimadzu Corporation, Canby, OR, USA) liquid chromatograph equipped with column Synergi polar RP 80A (150 × 4.6 mm, 5 μm). The chromatograph was equipped with a Shimadzu SPD-M20A detector (Shimadzu Corporation, Canby, OR, USA). Detection was carried out at a wavelength of λ = 240 nm. All chromatographic measurements were controlled by a CTO-10ASVP thermostat (Shimadzu Corporation, Canby, OR, USA). The eluent flow rate was 1.0 mL/min. Extracts were injected into the columns using the Rheodyne 20 μL injector. The DAD detector was set in the 200–800 nm range. Data acquisition and processing were carried out with a LabSolutions software (Shimadzu Corporation, Kyoto, Japan). The mobile phase was composed out of 0.04 M 1-butyl-3-methylimidazolium tetrafluoroborate in water (solvent A) and 1-butyl-3-methylimidazolium tetrafluoroborate in acetonitrile (solvent B). The following gradient elution was applied: 0–20 min, 25% B; 20–30 min, 25–32% B; 30–40 min, 32–40% B, 40–60 min, 40% B. Flow rate was 1 mL/min.

### 3.2. LC–QqQ–ESI–MS/MS Parameters

Identification of studied alkaloids in plant extracts (Table S1) was carried out using a triple quadrupole mass spectrometer (LC-MS/MS 8050 Shimadzu (Kyoto, Japan)) equipped with LabSolution version 5.8 software for data collection and instrumental control.

ESI-MS/MS spectrometer was coupled with UHPLC system (LC-30AD binary solvent delivery system, SIL-30AC autosampler and CTO-20AC thermostat) (Kyoto, Japan). Electrospray ionization (ESI+) was applied in the positive ion mode. The optimization of different MS parameters on the selectivity and MS response (multiple reaction monitoring, MRM peak areas) for the studied compounds was carried out without a chromatographic column. The optimal parameters were as follows: interface temperature 295 °C, DL temperature 260 °C, nebulizing gas flow 3 L/min, heating gas flow 9 L/min, and temperature of drying gas 375 °C. The studied alkaloids were monitored in the scheduled multiple reaction monitoring (MRM) mode. The total dwell time was 0.7 s. Chromatographic XDB-C18 column (4.6 mm × 50 mm, 1.8 µm, Agilent Technologies, Germany) was maintained at 25 ± 0.5 °C. The injected sample volume was 10 µL, while the mobile phase was composed ACN + 0.1% HCOOH (25:75, *v*/*v*) dosed at a flow rate of 0.3 mL/min. Addition of 0.1% formic acid to the acetonitrile/water mobile phase could improve peak shapes and increase MS detection sensitivity. All experiments were performed in triplicates. The data acquisition and processing were carried out using LabSolution Workstation software. The data were further processed using Microsoft Excel.

### 3.3. Chemicals and Plant Materials

Acetonitrile (MeCN), methanol (MeOH), 1-butyl-3-methylimidazolium tetrafluoroborate, diethylamine, ammonium acetate and acetic acid of chromatographic quality were obtained from E. Merck (Darmstadt, Germany). Acetylthiocholine iodide, 5,5’-dithiobis-(2-nitrobenzoic acid), TRIS hydrochloride, mercaptoethanol, acetylcholinesterase and butyrylcholinesterase were purchased from Sigma-Aldrich (St. Louis, MO, USA).

Alkaloid standards sanguinarine, chelerythrine and protopine were purchased from Chem Faces Biochemical Co., Ltd. (Wuhan, China).

Plant material was collected and identified in the Botanical Garden of Maria Curie-Skłodowska University in Lublin (Poland) in May, July, and September 2021. The plant material was collected from the same plants in various harvesting times.

Plants were divided into roots and aboveground parts. Aerial parts contained whole shoots with stem, leaves, and inflorescences (in material collected during flowering). Plants organs were cut into pieces and dried at ambient temperature for 1–2 weeks. Branches of *B. vulgaris* were decorticate and dried for 1–2 weeks under the same conditions. After grinding and mixing of the collected plant material, 5 g of the raw material was weighed.

### 3.4. Extraction Procedure

Weighted samples (5 g) of each plant material were macerated with 100 mL ethanol for 72 h and continuously extracted in an ultrasonic bath for 5 h. Extracts were filtered, the solvent evaporated under vacuum, and the residues dissolved in 30 mL of 2% sulfuric acid and defatted with diethyl ether (3 × 40 mL). The aqueous layers were subsequently basified with 25% ammonia to a pH of 9.5–10 and the alkaloids extracted with chloroform (3 × 50 mL). After evaporation of the organic solvent, the dried extracts were dissolved in 5 mL MeOH prior to HPLC analysis. Determination of berberine, protopine and chelidonine was performed after dissolving dried extracts prepared in the same manner in 0.5 mL MeOH.

### 3.5. Determination of Acetylcholinesterase Inhibitory Activity

For determination of acetylcholinesterase inhibitory activity of alkaloid standards and plant extracts by HPLC analysis was performed on the same apparatus as determination of alkaloid contents on Polar RP column with mobile phase A containing methanol and 0.0025 M diethylamine, mobile phase B containing acetate buffer at pH 3.8 and 0.0025 M diethylamine, mobile phase in gradient elution: 0–15 min, 10% A; 15–30 min, 10–70% A; 30–60 min, 70% A. Flow rate was 1 mL/min.

Stock solutions of acetylcholinesterase at concentration 200 U/mL and 2 U/mL, acetylthiocholine iodide at concentration 0.0125 M and 5′-dithiobis-(2-nitrobenzoic acid) at concentration 0.00167 M were prepared. Samples for determination of acetylcholinesterase inhibitory activity were prepared by addition of 50 µL of acetylthiocholine iodide, 50 µL of 5′-dithiobis-(2-nitrobenzoic acid), 50 µL of acetylcholinesterase at concentration 2 U/mL and phosphate buffer at pH 7.8 to obtaining 500 µL of final solution. Final concentration of acetylcholinesterase was 0.2 U/mL for determination of IC_50_ values. All components were mixed and samples were incubated for 15 min at 37 °C. Samples were filtrated by syringe filter (CHROMAFIL Xtra, PVDF-29/25 0.20 µm) and injected into HPLC system. Detection was performed at λ = 405 nm. All experiments were performed in triplicates.

### 3.6. Determination of Butyrylcholinesterase Inhibitory Activity

For determination of butyrylcholinesterase inhibitory activity of alkaloid standards and plant extracts by HPLC analysis was performed on the same apparatus, column and mobile phase as determination of acetylcholinesterase inhibitory activity.

Stock solutions of butyrylcholinesterase at concentration 200 U/mL and 2 U/mL, butyrylthiocholine iodide at concentration 0.0125 M and 5′-dithiobis-(2-nitrobenzoic acid) at concentration 0.00167 M were prepared. Samples for determination of butyrylcholinesterase inhibitory activity were prepared by addition of 50 µL of butyrylthiocholine iodide, 50 µL of 5′-dithiobis-(2-nitrobenzoic acid), 50 µL of butyrylcholinesterase at concentration 2 U/mL and phosphate buffer at pH 7.8 to obtaining 500 µL of final solution. Final concentration butyrylcholinesterase was 0.2 U/mL for determination of IC_50_ values. All components were mixed and samples were incubated for 15 min at 37 °C. Samples were filtrated by syringe filter (CHROMAFIL Xtra, PVDF-29/25 0.20 µm) and injected into HPLC system. Detection was performed at λ = 405 nm. All experiments were performed in triplicates.

### 3.7. Kinetic Analysis of the Cholinesterase Inhibition

Different concentrations of acetylthiocholine iodide (0.125, 0.25, 0.625 and 0.9375 mM) were used to construct reciprocal plots of 1/V versus 1/[S] to estimate the most active alkaloids sanguinarine and chelerythrine inhibition constant/inhibition constant (Ki). For determination of 5-thio-2-nitro-benzoic acid, the product of reaction between the thiocholine with Ellman’s reagent, HPLC-DAD method was applied. The chromatographic analysis was performed in the same conditions as was used for determination of cholinesterase activity described above. Quantification of 5-thio-2-nitro-benzoic acid was performed by a calibration curve method. 5-thio-2-nitrobenzoic acid was prepared from 5′-dithiobis-(2-nitrobenzoic acid) as described by Landino et al. [41]. 0.5 g of 5′-dithiobis-(2-nitrobenzoic acid) in 25 mL 0.5 M Tris–HCl, pH 8.8, was treated with 2.5 mL β-mercaptoethanol. The pH of the solution was adjusted to 1.5 with 6M HCl. 5-thio-2-nitro-benzoic acid was formed after 6–8 h at 4 °C. The crystals of -thio-2-nitro-benzoic acid were filtered and washed with cold 0.1 M HCl. Types of inhibition were determined by the graphical analysis of Lineweaver–Burk plots and its secondary plots.

### 3.8. Molecular Docking Method

Molecular docking is an important tool to search for and analyze the interactions occurring between the receptor protein and the molecule acting as ligand (http://dx.doi.org/10.1016/j.molstruc.2016.06.0510022-2860/, © 2016 Published by Elsevier B.V.). The receptor structures of AChE (id: 4m0e) and BChE (id: 1p0p) were downloaded in pdb format via the RCSB PDB website (https://www.rcsb.org/, accessed on 13 May 2022). Using Molegro Molecular Viewer 7 software (http://molexus.io/molegro-molecular-viewer/, accessed on 13 May 2022), the extracted structures were cleaned, i.e., water molecules, attached ligands and cofactors were removed. The ligand structures were downloaded from PubChem database (https://pubchem.ncbi.nlm.nih.gov/, accessed on 13 May 2022.) in 3D format and sfd extension.

Next, using the OpenBabel program (http://openbabel.org/wiki/Main_Page, accessed on 13 May 2022), the sfd format was converted to pdb format without loss of quality of the structures. The receptor and ligand structures were then optimized by lowering their energy minimum in the Molecular Operating Environment program (https://www.chemcomp.com/, accessed on 13 May 2022). The ligand attachment sites were also determined for the prepared receptors. The last step, molecular docking, was performed using AutoDock 4.2.6 (https://autodock.scripps.edu/, accessed on 13 May 2022) according to the procedure described in Dyrda-Terniuk (Int. J. Mol. Sci. 2022, 23, 2940. https://doi.org/10.3390/ijms23062940) at 298.15 K. The obtained structures after the molecular docking step were visualized using Protein-Ligand Interaction Profiler (https://plip-tool.biotec.tu-dresden.de/plip-web/plip/index, accessed on 13 May 2022).

## 4. Conclusions

Ellman’s method is significantly limited when the measurement is performed in solutions containing substances that exhibit adsorption at a wavelength of about λ = 412 nm, such as many plant extracts. The application of the HPLC-DAD method for determination of acetylcholinesterase and butyrylcholinesterase inhibition activity enables avoiding errors related to the overlapping adsorption of 5-thio-2-nitro-benzoic acid and some components of plant extracts. In the literature, there are only several reports on in vitro determination of cholinesterases, especially butyrylcholinesterase inhibition activity by chromatographic methods. There are also no reports on the optimization of conditions for the determination of butyrylcholinesterase inhibition by HPLC-DAD.

The highest content of all the investigated alkaloids was determined in the plant materials collected in May and the content was decreased in the plant materials collected in July and September. A higher content of protopine was determined in roots compared to the content in aerial parts, while higher contents of sanguinarine and chelerythrine were determined in aerial parts.

All the investigated isoquinoline alkaloids exhibited cholinesterase inhibition activity. The highest activity was determined for chelerythrine with IC_50_ = 0.72 μM against acetylcholinesterase and 2.15 μM against butyrylcholinesterase. The IC_50_ values obtained by our procedure for galantamine were 1.46 μM against acetylcholinesterase and 6.31 μM against butyrylcholinesterase; for rivastigmine, the IC_50_ values were 10.23 and 8.38 μM against acetylcholinesterase and butyrylcholinesterase, respectively.

All the investigated extracts effectively inhibited the cholinesterase activity. An especially high activity was observed for the extracts obtained from the aerial parts collected in May. The obtained results showed that the extracts obtained from *Macleaya cordata* are promising as inhibitors of cholinesterase activity.

To the best of our knowledge, the acetylcholinesterase and butyrylcholinesterase inhibition activity of *Macleaya cordata* extracts from aerial parts collected in various months had not been investigated previously.

These results indicate a need for further in vitro and in vivo investigations of plant extracts obtained from *Macleaya cordata.* The investigated extracts and alkaloids contained therein may be developed as new candidates for treatment of neurodegenerative diseases, including at the later stages of Alzheimer’s disease.

## Figures and Tables

**Table 1:** Content of isoquinoline alkaloids in the plant extracts and the standard deviation of these values.

Time of Plant Collection	Part of the Plant	Protopine (mg/g of Dry Plant Material)	Sanguinrine (mg/g of Dry Plant Material)	Chelerythrine (mg/g of Dry Plant Material)
May	Aerial part	1.361 ± 0.181	4.091 ± 0.364	5.395 ± 0.489
	Root	16.576 ± 1.784	1.647 ± 0.125	1.105 ± 0.102
July	Aerial part	2.288 ± 0.211	2.888 ± 0.248	3.474 ± 0.375
	Root	7.658 ± 0.741	1.780 ± 0.153	1.544 ± 0.151
September	Aerial part	1.354 ± 0.128	2.413 ± 0.198	3.257 ± 0.309
	Root	8.693 ± 0.789	1.233 ± 0.105	0.883 ± 0.111

**Table 2:** Content of isoquinoline alkaloids in dry residue of plant extracts and standard deviation of these values.

Time of Plant Collection	Part of Plant	Dry Residue (mg/g of Dry Plant Material)	Protopine (mg/g of Dry Plant Extract)	Sanguinrine (mg/g of Dry Plant Extract)	Chelerythrine (mg/g of Dry Plant Extract)
May	Aerial part	10.61 ± 0.85	118.31 ± 15.74	372.08 ± 33.09	501.48 ± 45.46
	Root	32.34 ± 2.74	518.00 ± 55.74	51.47 ± 3.89	34.17 ± 3.15
July	Aerial part	12.07 ± 0.93	189.56 ± 17.48	239.27 ± 20.56	287.82 ± 31.06
	Root	26.08 ± 2.15	293.64 ± 28.41	68.25 ± 5.87	59.29 ± 5.80
September	Aerial part	7.87 ± 0.61	171.99 ± 16.26	306.61 ± 25.15	413.85 ± 39.27
	Root	26.98 ± 2.18	322.20 ± 29.23	45.70 ± 3.90	3.27 ± 0.41

**Table 3:** IC_50_ values obtained for alkaloid standards.

Alkaloid	IC_50_ values			
	Acetylcholinesterase		Butyrylcholinesterae	
	(μM)	(μg/mL)	(μM)	(μg/mL)
Chelerythrine	0.72 ± 0.08	0.25	2.15 ± 0.19	0.75
Sanguinarine	5.57 ± 0.47	1.85	9.82 ± 0.91	3.26
Proptopine	69.81 ± 4.20	24.67	52.84 ± 4.81	18.67
Galantamine	1.46 ± 0.13	0.42	6.31 ± 0.51	1.81
Rivastigmine	10.23 ± 0.93	2.56	8.38 ± 0.63	2.10

**Table 4:** IC_50_ values obtained for plant extracts.

Time of Plant Collection	Part of Plant	IC_50_ Values (μg/mL)	
		Acetylcholinesterase	Butyrylcholinesterase
May	Aerial part	9.61 ± 0.92	21.17 ± 1.91
	Root	53.80 ± 4.89	62.84 ± 5.10
July	Aerial part	17.42 ± 1.25	28.04 ± 1.98
	Root	42.28 ± 3.62	60.29 ± 4.90
September	Aerial part	12.94 ± 0.97	25.74 ± 1.78
	Root	59.14 ± 4.40	64.28 ± 5.18

**Table 5:** Equations of correlation curves and Pearson correlation coefficients (r), values of isoquinoline alkaloid contents and cholinesterase activity inhibition expressed as IC_50_ (µg/mL) of the investigated plant extracts.

Alkaloid	Acetylcholinesterase		Butyrylcholinesterase	
	Equation	r	Equation	r
Chelerythrine	y = −7.5822x + 502.48	0.9678	y = −10.076x + 657.25	0.9749
Sanguinarine	y = −5.1131x + 373.32	0.9702	y = −6.8244x + 478.97	0.9815
Protopine	y = 4.5094x + 98.952	0.8540	y = 5.9102x + 10.514	0.8485
Sum of chelerythrine and sanguinarine	y = −12.695x + 875.8	0.9691	y = −16.901x + 1136.2	0.9779
Sum of all alkaloids	y = −8.1859x + 974.76	0.8963	y = −10.99x + 1146.7	0.9121

**Table 6:** Predicted binding sites for AChE.

Site	Size	Score	Residues
1	133	3.22	1:(ASN233 GLY234 PRO235 TRP236 ALA237 THR238 VAL239 GLY240 GLU243 ARG247 LEU289 PRO290 GLN291 SER293 ARG296 PHE297 VAL300 VAL303 THR311 GLU313 ASN317 VAL367 PRO368 GLN369 VAL370 ASP404 HIS405 CYS409 PRO410 GLN413 TRP532 ASN533 LEU536 PRO537 LEU540)
2	55	1.34	1:(ASP74 THR83 TRP86 ASN87 GLY120 GLY121 GLY122 TYR124 SER125 TYR133 GLU202 SER203 TRP236 PHE295 PHE297 TYR337 PHE338 TYR341 HIS447 GLY448 ILE451)
3	25	0.14	1:(ARG475 TYR479 GLU491 PRO492 ALA497 PRO498 GLN499 PRO517 LEU518)
4	39	0.13	1:(TYR72 VAL73 ASP74 THR75 LEU76 TRP286 LEU289 GLN291 GLU292 SER293 ARG296 TYR341 GLY342)
5	48	0.07	1:(ALA412 GLN413 GLY416 ARG417 TYR503 THR504 ALA505 GLN508 ALA526 CYS529 ALA530 ASN533 ARG534)
6	27	−0.07	1:(GLU81 GLU84 MET85 TRP86 ASN87 PRO88 ASN89 LEU130 ASP131 VAL132 ARG463)
7	17	−0.28	1:(VAL12 ARG13 GLY14 ILE35 PRO36 PHE37 LYS53 TRP56 TRP182 ASN186)
8	12	−0.4	1:(ASP131 ASP134 ARG136 PHE137 ILE457 ASP460 SER462 ARG463)
9	13	−0.45	1:(LEU213 SER215 PRO216 ARG219 ASP320 PHE321 HIS322 LEU324)
10	19	−0.45	1:(ARG13 PHE37 PRO52 LYS53 LEU178 GLN181 TRP182 GLU185 ASN186)
11	16	−0.6	1:(LYS23 THR24 PRO25 ARG136 PHE137 LEU459 ASP460 PRO461)
12	10	−0.6	1:(LEU213 LEU214 SER215 PRO216 SER309 LEU315 GLY319 ASP320 PHE321)
13	7	−0.64	1:(PRO104 PRO108 PRO111 THR112 PRO113 ARG143)
14	6	−0.7	1:(LYS332 VAL429 GLU431 TYR510 ARG521 LEU524 ARG525)
15	11	−0.72	1:(PHE65 GLN66 SER67 ARG90 GLU91)

**Table 7:** AChE and BChE: free energy of binding, inhibition constant, and bonds lengths.

Receptor	Ligand	Estimated Free Energy of Binding (kcal/mol)	Estimated Inhibition Constant Ki (μM)	Hydrophobic Interactions (length; A)	Hydrogen Bonds (Length; A)	Salt Bridge (Length; A)	π-Stacking (Length; A)
AChE (4m0e)	Chelerythrine	−7.5	3.17	5 (3.56, 3.57, 3.63, 3.24, 3.93)	1 (3.69)	0	0
	Protopine	−6.51	16.99	3 (3.44, 3.39, 3.51)	1 (2.88)	1 (5.01)	0
	Sanguinarine	−7.5	3.14	4 (3.68, 3.56, 3.70, 3.24)	1 (3.75)	0	0
BChE (1p0p)	Chelerythrine	−8.18	1.01	3 (3.99, 3.40, 3.88, 3.49)	3 (3.80, 3.06, 3.30)	0	0
	Protopine	−8.36	0.75	1 (3.40)	3 (4.02, 2.95, 3.68)	1 (4.54)	1 (4.99)
	Sanguinarine	−8.14	1.06	3 (3.61, 3.67, 3.25)	2 (3.11, 2.63)	0	0

**Table 8:** Predicted binding sites for butyrylcholinesterase.

Site	Size	PLB	Residues
1	132	2.7	1:(ASN68 ILE69 ASP70 GLN71 SER72 GLY78 SER79 TRP82 ASN83 TYR114 GLY115 GLY116 GLY117 THR120 GLY121 TYR128 GLU197 SER198 ALA199 TRP231 PRO285 LEU286 SER287 VAL288 ALA328 PHE329 TYR332 PHE398 TRP430 MET437 HIS438 GLY439 TYR440 ILE442)
2	70	2.12	1:(PRO230 TRP231 GLU238 ARG242 GLU276 ALA277 PHE278 VAL280 PRO281 TYR282 GLY283 THR284 LEU286 SER287 VAL288 ASN289 PHE357 PHE358 PRO359 GLY360 VAL361 TYR396)
3	60	0.57	1:(ASN228 ALA229 PRO230 ASP304 LEU307 GLU308 ASP395 TYR396 ASN397 CYS400 PRO401 GLU404 LYS408 TRP522 THR523 PHE526 PRO527)
4	26	−0.04	1:(LYS9 ASN10 TYR33 SER48 LEU49 LEU173 GLN176 TRP177 LYS180 ASN181)
5	28	−0.1	1:(CYS400 LEU403 GLU404 LYS407 GLN498 LEU514 ARG515 ALA516 CYS519 ARG520 THR523 SER524)
6	27	−0.28	1:(HIS372 TYR373 THR374 ASP375 TRP376 ALA388 ASP391 ARG515 GLN517 GLN518)
7	15	−0.46	1:(SER368 PHE371 HIS372 ARG520 PHE521 PHE525)
8	17	−0.46	1:(ILE462 LEU463 SER466 ASN485 SER487 ASN504 THR505 GLU506 SER507 THR508)
9	9	−0.47	1:(HIS77 MET81 ASN85 LEU125 HIS126 VAL127)
10	23	−0.47	1:(GLN67 ASN68 ILE69 GLN71 GLN270 LEU273 LEU274 ALA277)
11	22	−0.55	1:(LYS45 PRO46 PHE169 GLN172 LEU173 GLN176 ASP297)
12	14	−0.57	1:(GLN351 GLU352 LEU354 LYS355 GLU363 LYS366)
13	8	−0.61	1:(THR19 HIS126 LYS131 PHE132 GLU451 ASP454)
14	21	−0.66	1:(PHE21 VAL136 LEU450 ARG452 GLU461 ARG465)
15	12	−0.72	1:(LEU450 LYS458 GLU461 ILE462 ARG465 ASN485)

## Data Availability

Not applicable.

## Supplementary Materials

The following supporting information can be downloaded at: https://www.mdpi.com/article/10.3390/molecules27113606/s1, Figure S1: (A) The dose of chelerythrine response curve of the acetylcholinesterase inhibitory activity. (B) The dose of sanguinarine response curve of the acetylcholinesterase inhibitory activity. (C) The dose of protopine response curve of the acetylcholinesterase inhibitory activity. (D) The dose of chelerythrine response curve of the butyrylcholinesterase inhibitory activity. (E) The dose of sanguinarine response curve of the butyrylcholinesterase inhibitory activity. (F) The dose of protopine response curve of the butyrylcholinesterase inhibitory activity.; Figure S2: (A) The dose of extract obtained from Macleaya cordata aerial part collected in May response curve of the acetylcholinesterase inhibitory activity. (B) The dose of extract obtained from Macleaya cordata root collected in May response curve of the acetylcholinesterase inhibitory activity. (C) The dose of extract obtained from Macleaya cordata aerial part collected in July response curve of the acetylcholinesterase inhibitory activity. (D) The dose of extract obtained from Macleaya cordata root collected in July response curve of the acetylcholinesterase inhibitory activity. (E) The dose of extract obtained from Macleaya cordata aerial part collected in September response curve of the acetylcholinesterase inhibitory activity. (F) The dose of extract obtained from Macleaya cordata root collected in September response curve of the acetylcholinesterase inhibitory activity.; Figure S3: (A) The dose of extract obtained from Macleaya cordata aerial part collected in May response curve of butyrylcholinesterase inhibitory activity. (B) The dose of extract obtained from Macleaya cordata root collected in May response curve of butyrylcholinesterase inhibitory activity. (C) The dose of extract obtained from Macleaya cordata aerial part collected in July response curve of butyrylcholinesterase inhibitory activity. (D) The dose of extract obtained from Macleaya cordata root collected in July response curve of butyrylcholinesterase inhibitory activity. (E) The dose of extract obtained from Macleaya cordata aerial part collected in September response curve of butyrylcholinesterase inhibitory activity. (F) The dose of extract obtained from Macleaya cordata root collected in September response curve of butyrylcholinesterase inhibitory activity.; Table S1: MS parameters and conditions used for determination and identification of selected alkaloids in plant extract samples.
