# Supplementary material for: Isoquinoline Alkaloid Contents in Macleaya cordata Extracts and Their Acetylcholinesterase and Butyrylcholinesterase Inhibition
Source: Molecules. 2022 Jun 3;27(11):3606. doi: 10.3390/molecules27113606 (PMC9182188; doi:10.3390/molecules27113606)
Supplement: Supplementary file 1 [file molecules-27-03606-s001.zip › molecules-1728387-supplementary.pdf]

Table S1. MS parameters and conditions used for determination and identification of selected alkaloids in plant extract samples.

Compound	Elemental composition	t_R (min)	Polarity	Precursor ion (*m/z*)	MS/MS
Allocryptopine	$C_{21}H_{24}NO_5$ $[M+H]^+$	3.23	ESI+	370.15	352.70
					336.30
					321.10
					306.20
					290.15
					275.10
					206.05
					189.10
					188.05
					181.10
					165.10
					149.05
Chelerythrine	$C_{21}H_{18}NO_4$ $[M+H]^+$	3.98	ESI+	348.20	332.10
					303.95
					290.15
					274.60
					244.30
					231.60
Protopine	$C_{20}H_{20}NO_5$ $[M+H]^+$	1.68	ESI+	354.30	334.10
					307.60
					274.95
					246.50
					205.70
					189.05
					173.50
					146.90
Sanguinarine	$C_{20}H_{14}NO_4$ $[M+H]^+$	2.75	ESI+	332.10	316.60
					304.20
					301.75
					288.65
					274.20
					246.05
					216.20
					150.90

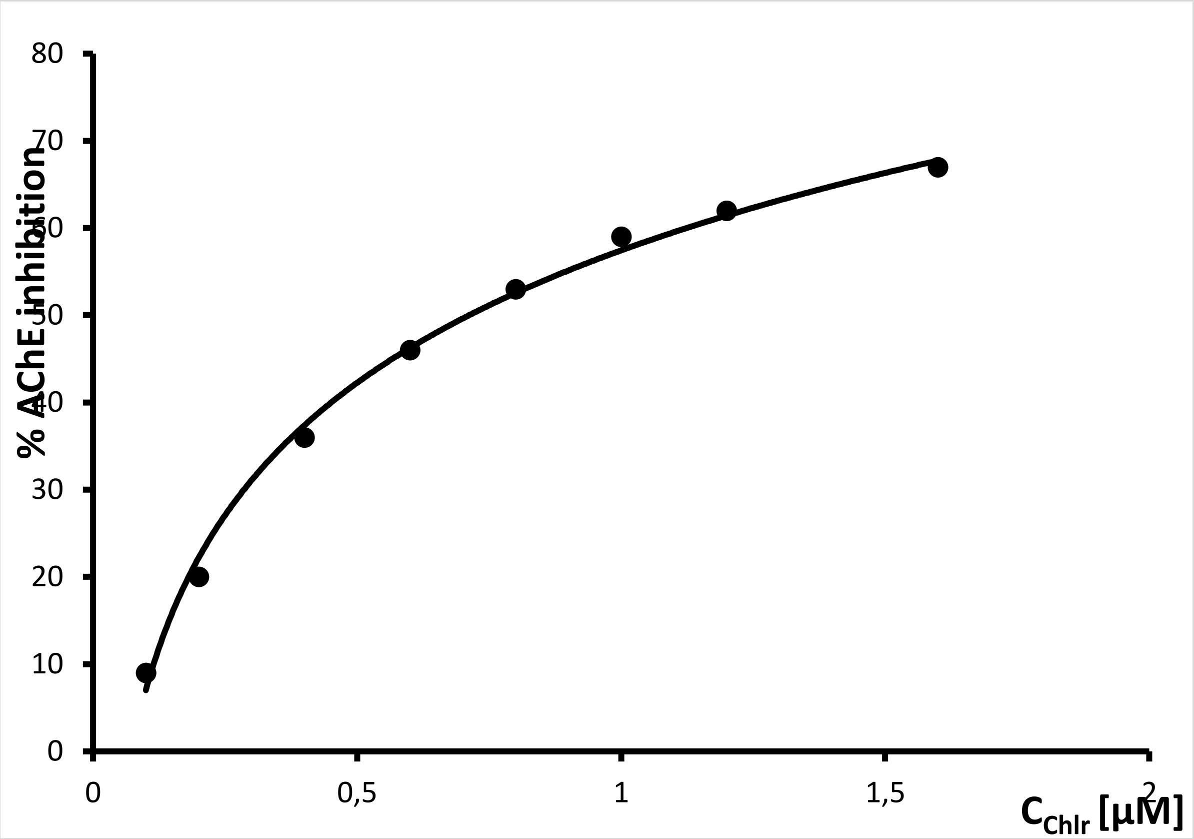

Figure S1A. The dose of chelerythrine response curve of the acetylcholinesterase inhibitory activity.

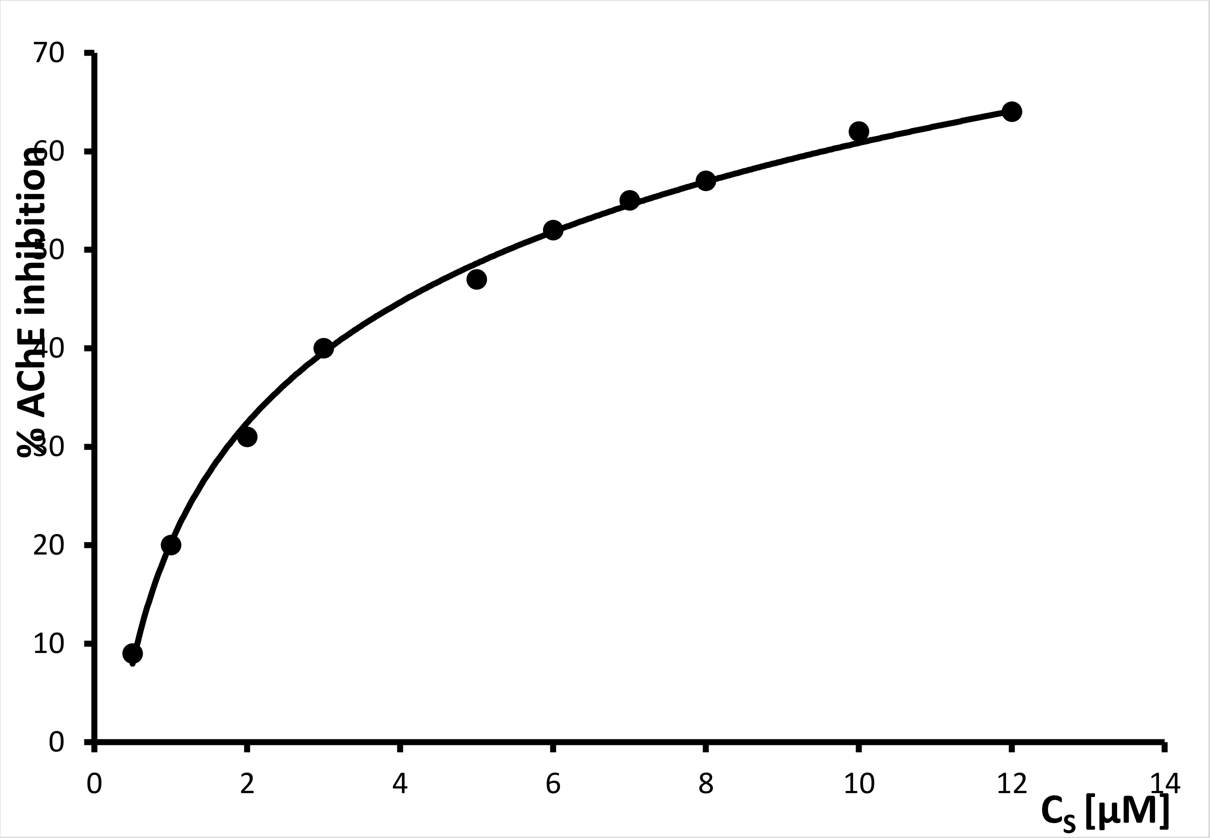

Figure S1B. The dose of sanguinarine response curve of the acetylcholinesterase inhibitory activity.

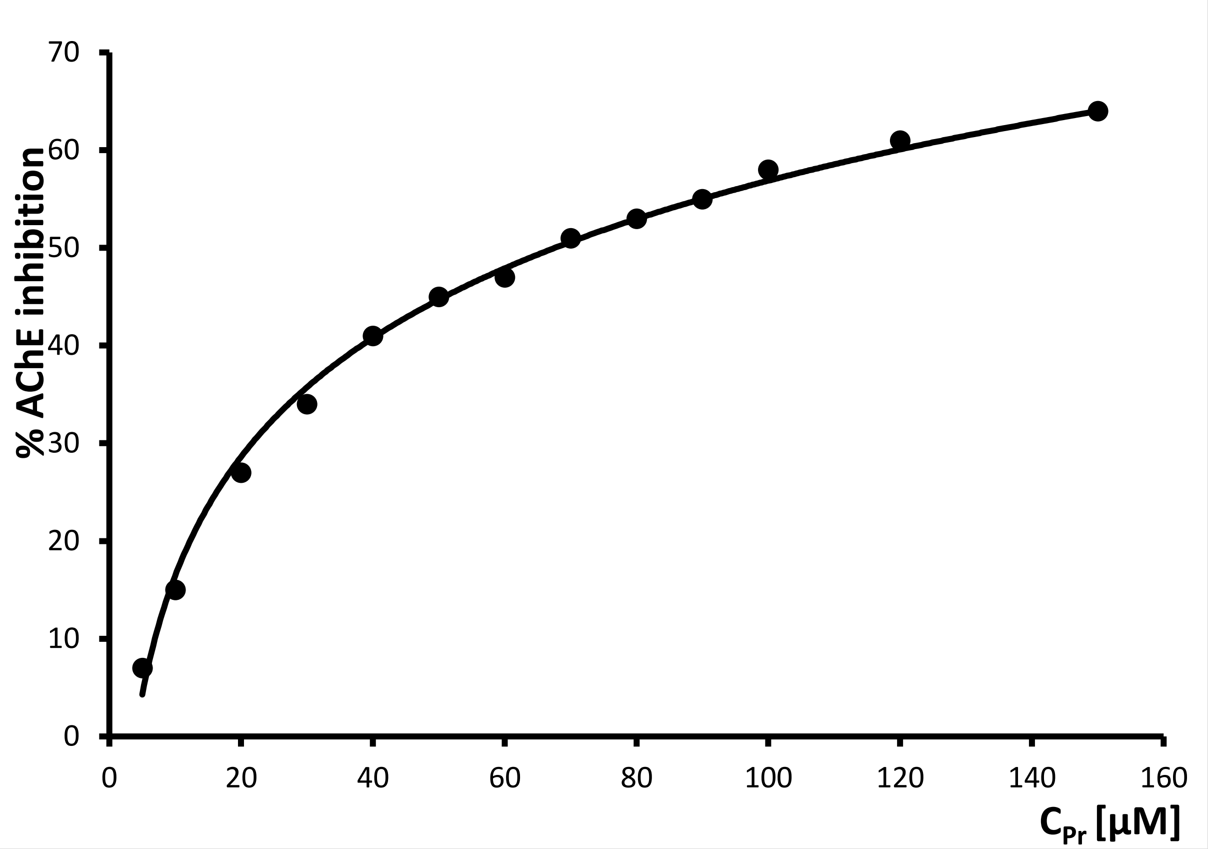

Figure S1C. The dose of protopine response curve of the acetylcholinesterase inhibitory activity.

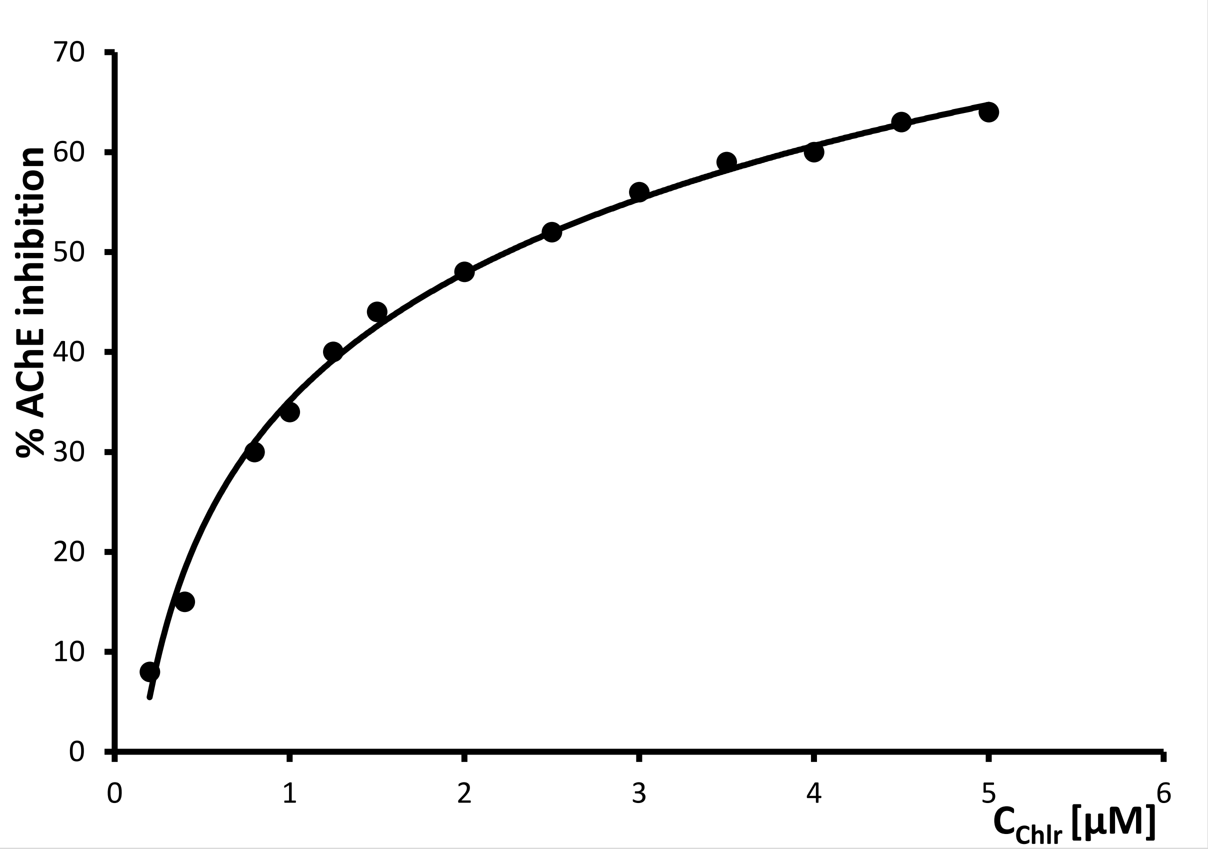

Figure S1D. The dose of chelerythrine response curve of the butyrylcholinesterase inhibitory activity.

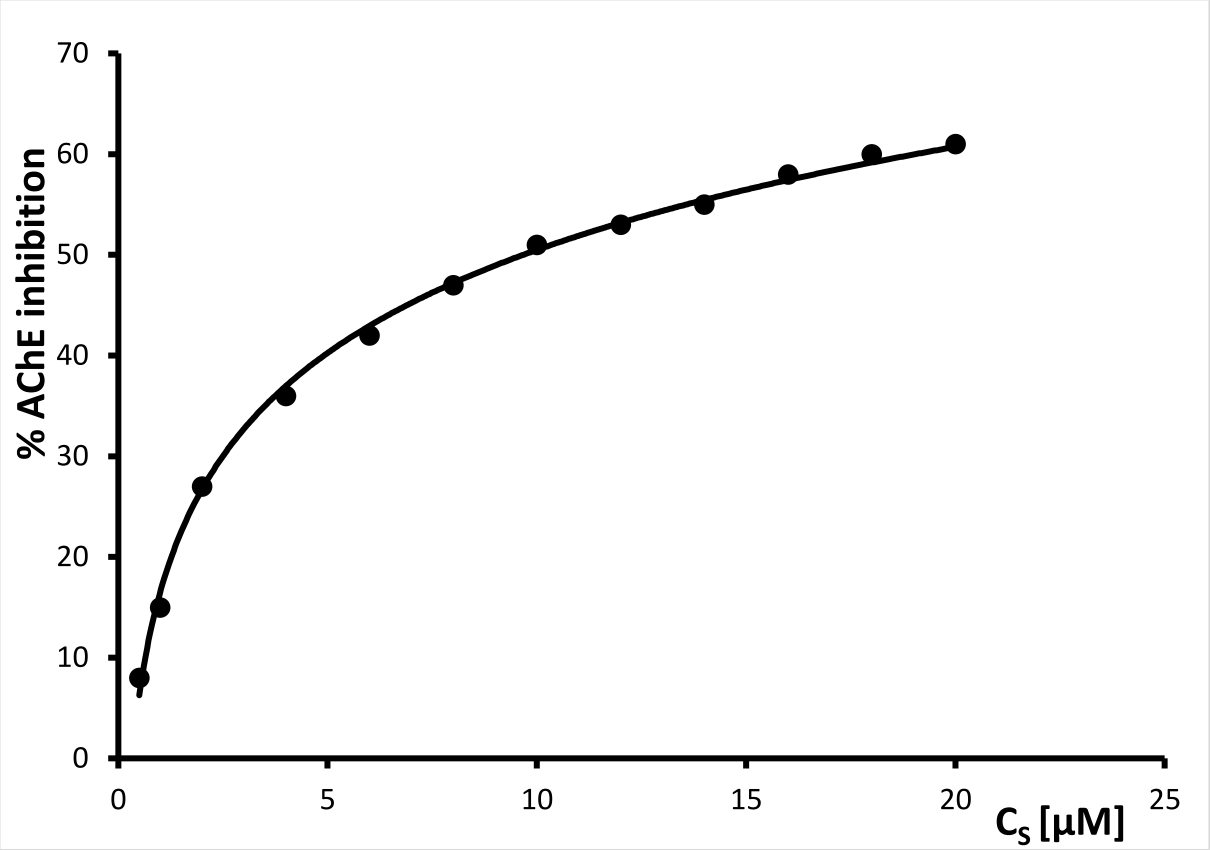

Figure S1E. The dose of sanguinarine response curve of the butyrylcholinesterase inhibitory activity.

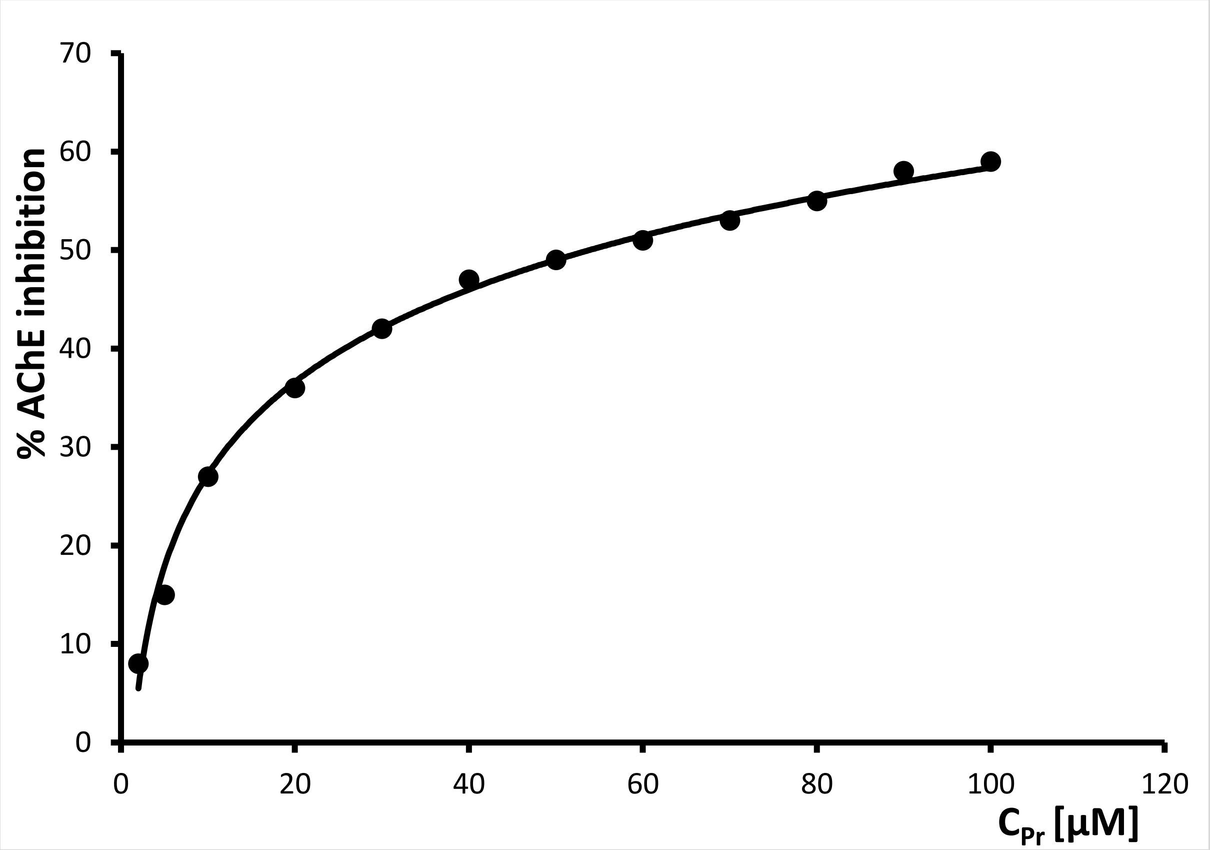

Figure S1F. The dose of protopine response curve of the butyrylcholinesterase inhibitory activity.

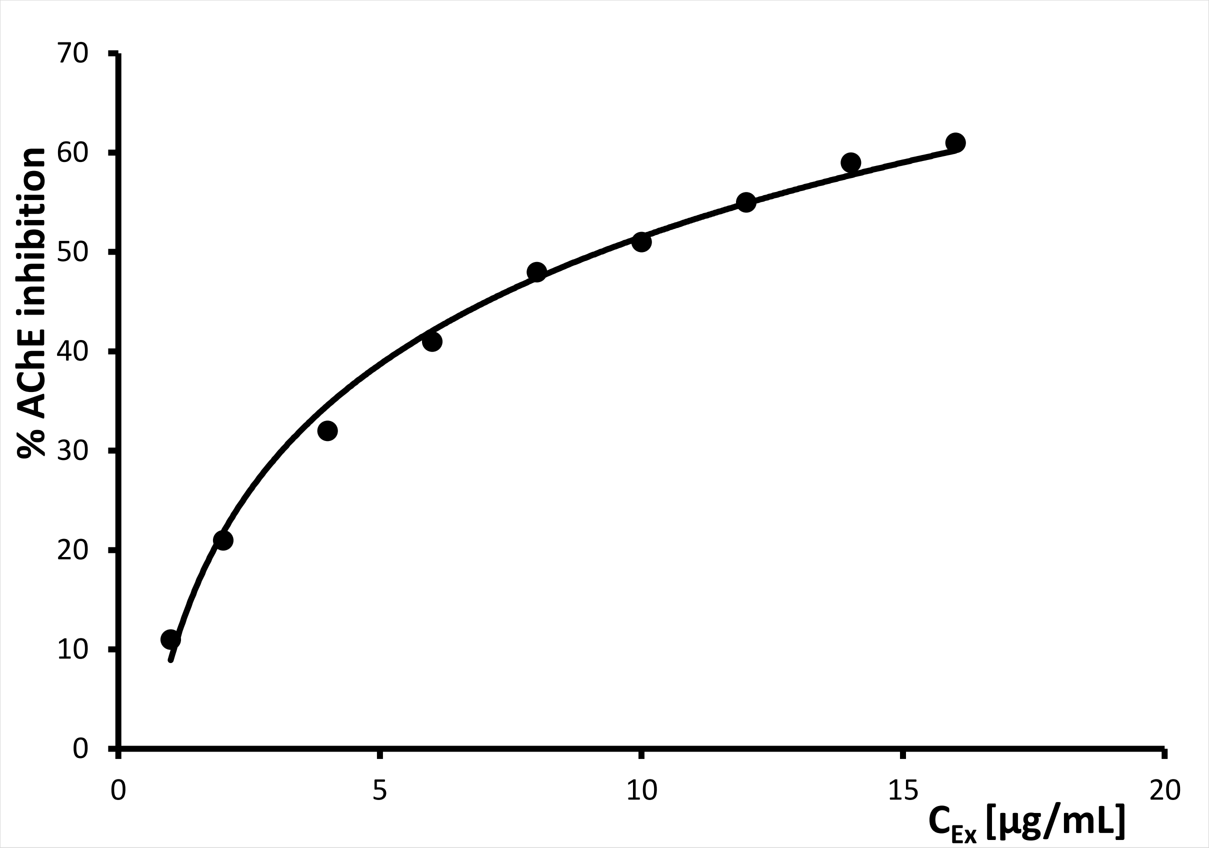

Figure S2A. The dose of extract obtained from *Macleaya cordata* aerial part collected in May response curve of the acetylcholinesterase inhibitory activity.

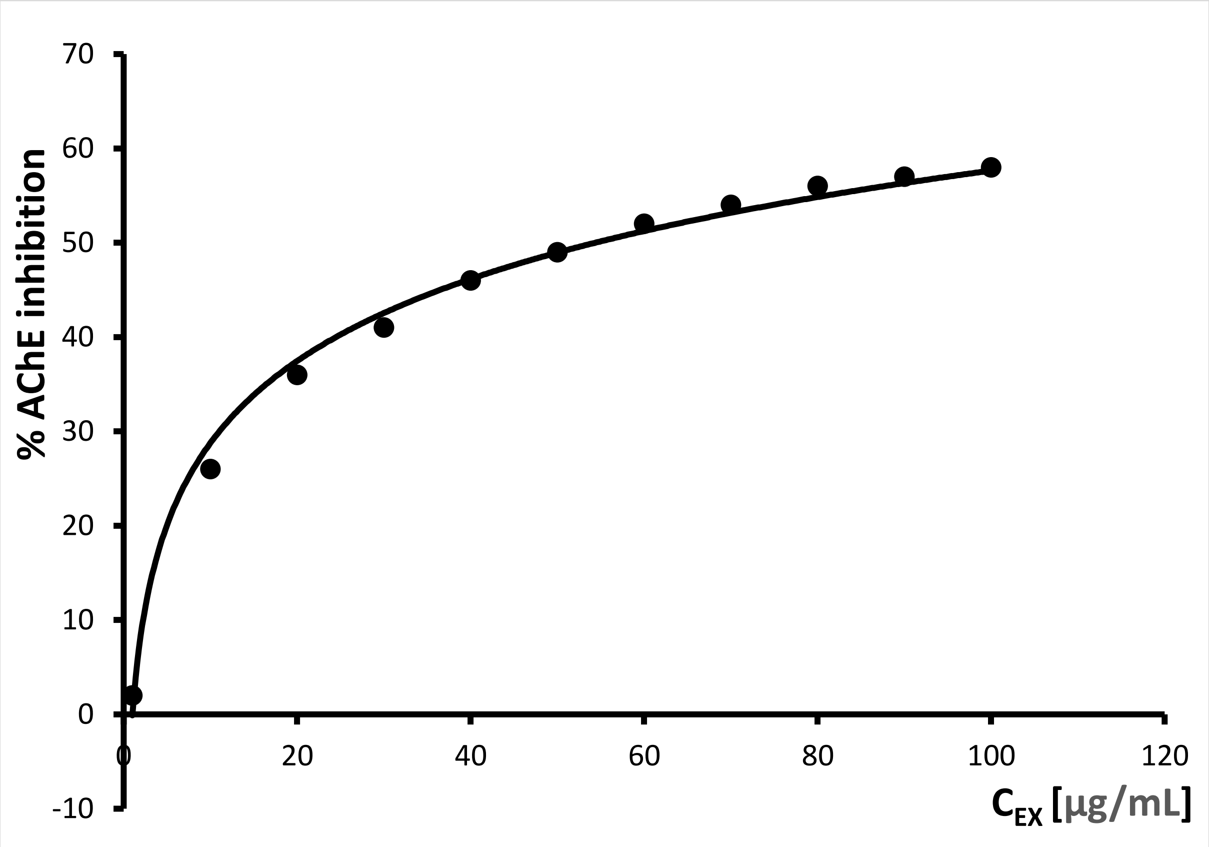

Figure S2B. The dose of extract obtained from *Macleaya cordata* root collected in May response curve of the acetylcholinesterase inhibitory activity.

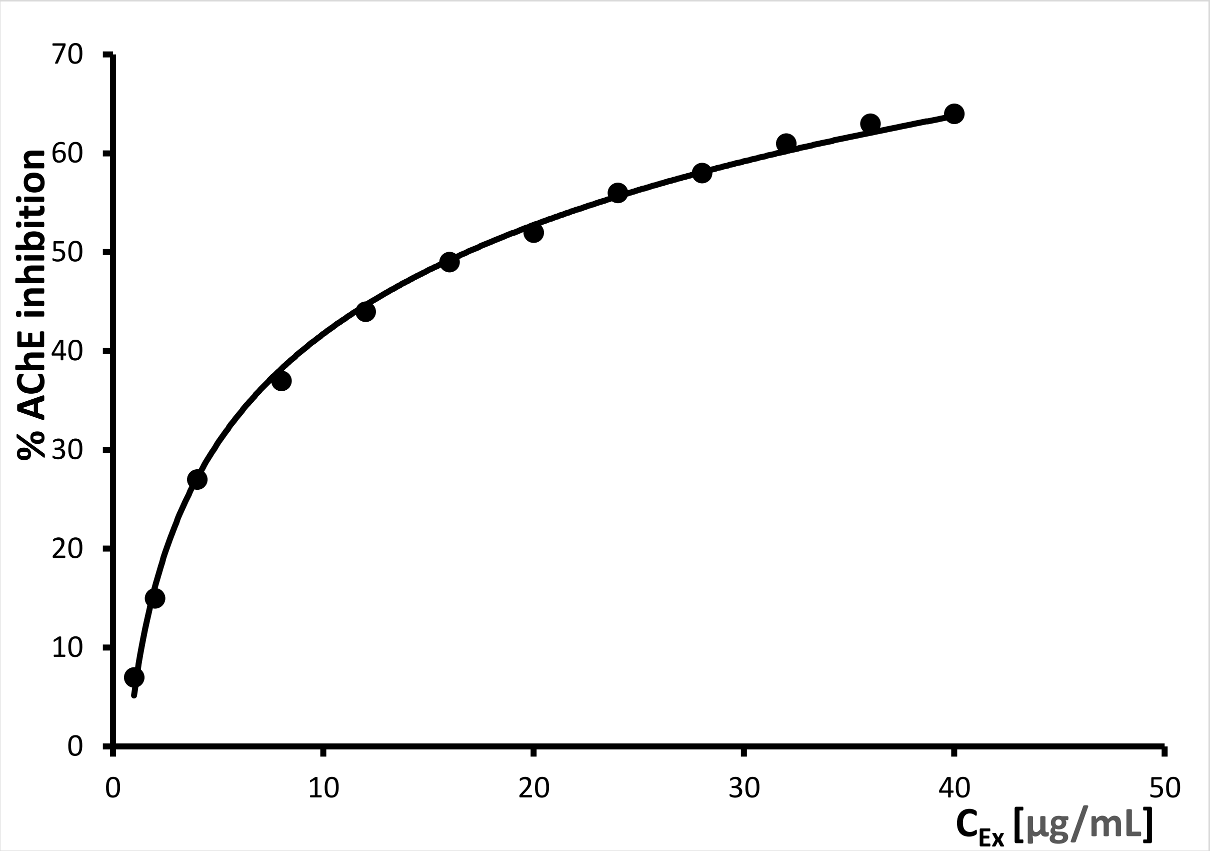

Figure S2C. The dose of extract obtained from *Macleaya cordata* aerial part collected in July response curve of the acetylcholinesterase inhibitory activity.

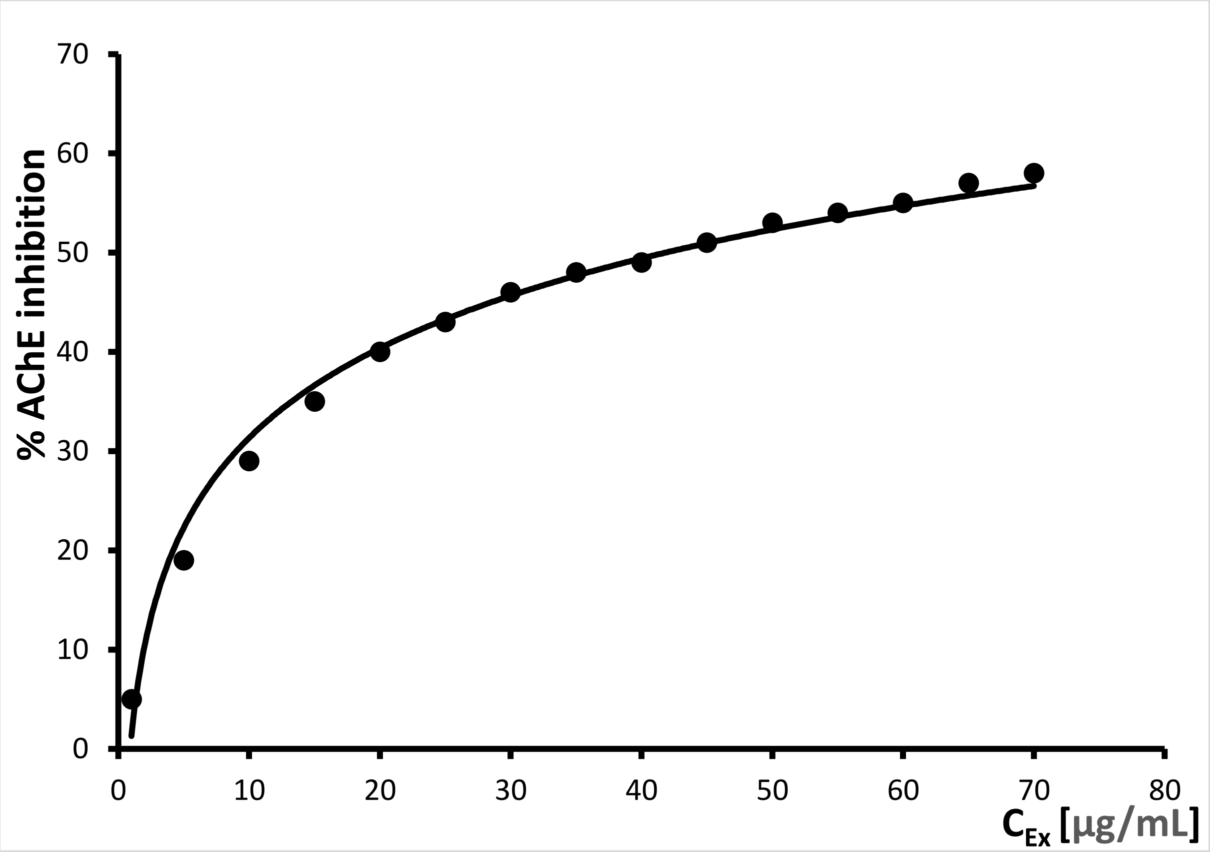

Figure S2D. The dose of extract obtained from *Macleaya cordata* root collected in July response curve of the acetylcholinesterase inhibitory activity.

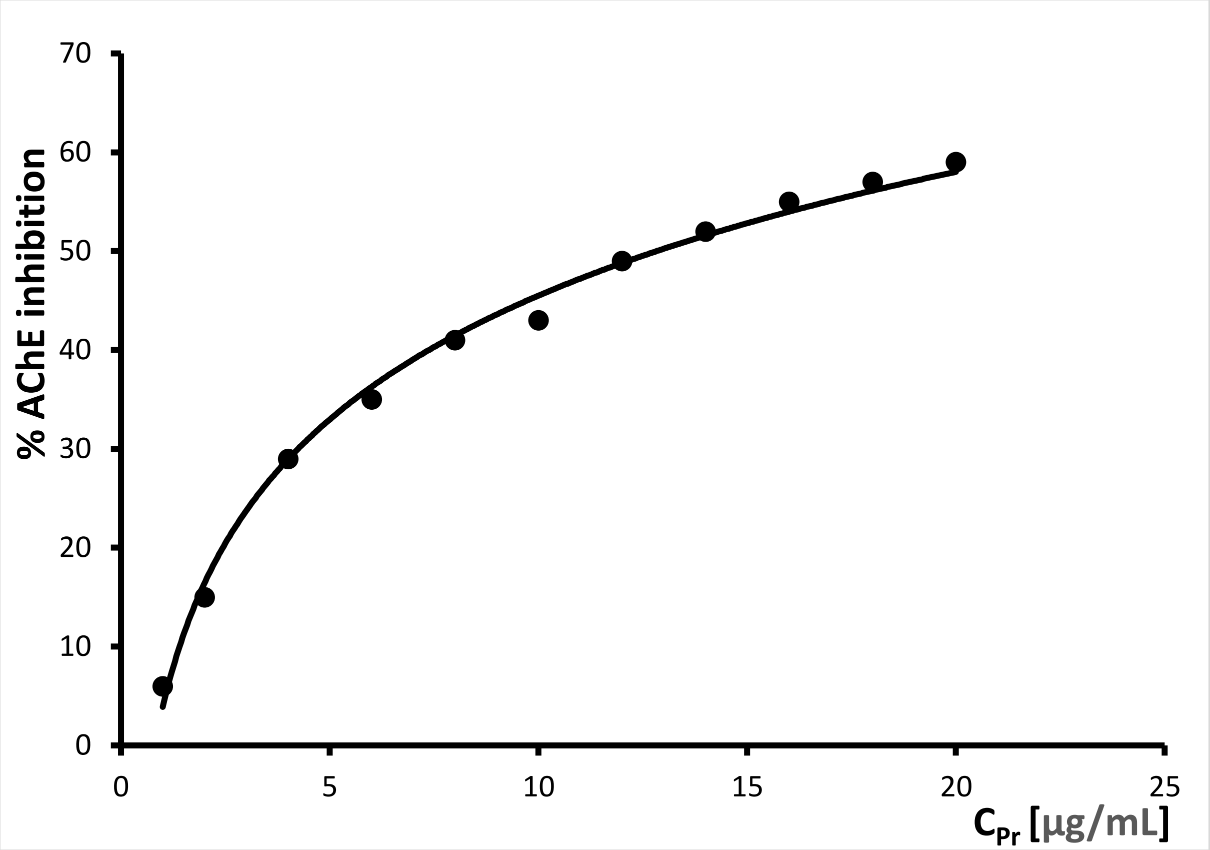

Figure S2E. The dose of extract obtained from *Macleaya cordata* aerial part collected in September response curve of the acetylcholinesterase inhibitory activity.

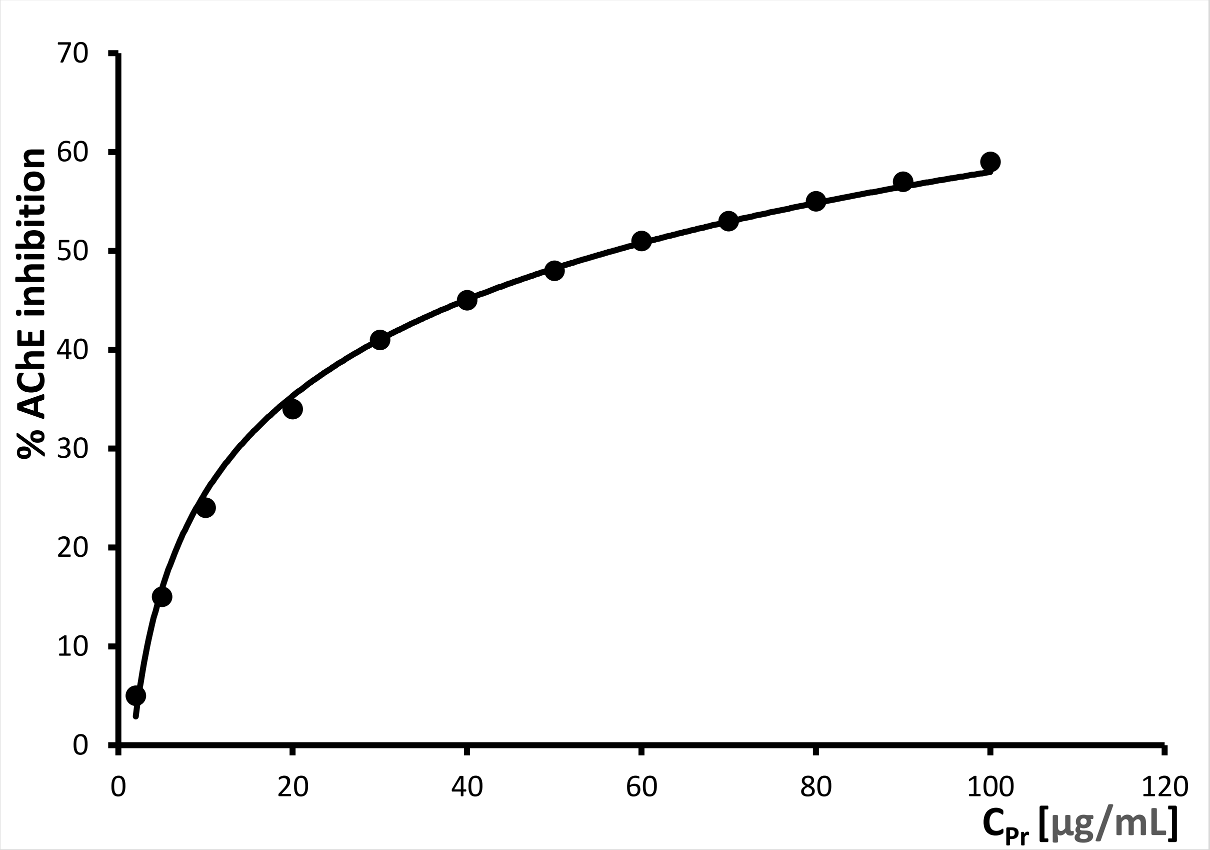

Figure S2F. The dose of extract obtained from *Macleaya cordata* root collected in September response curve of the acetylcholinesterase inhibitory activity.

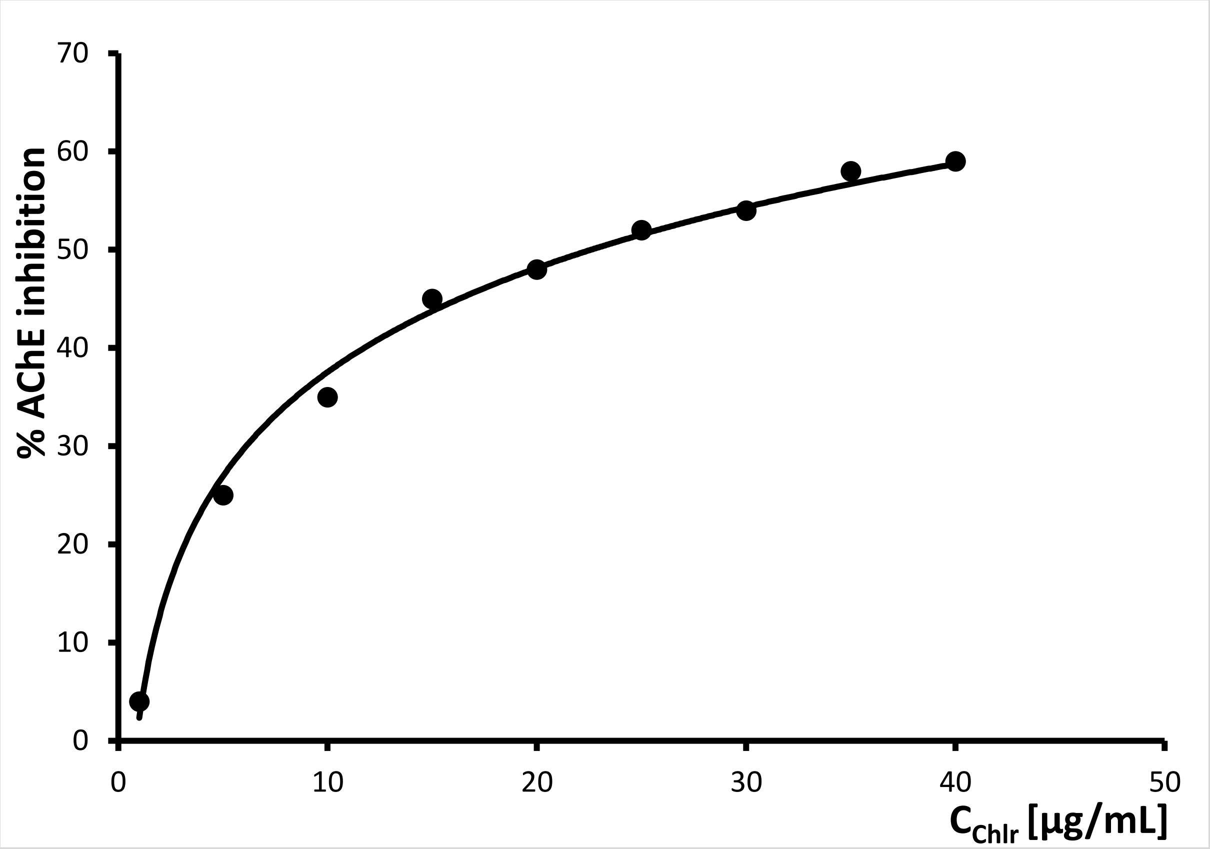

Figure S3A. The dose of extract obtained from *Macleaya cordata* aerial part collected in May response curve of butyrylcholinesterase inhibitory activity.

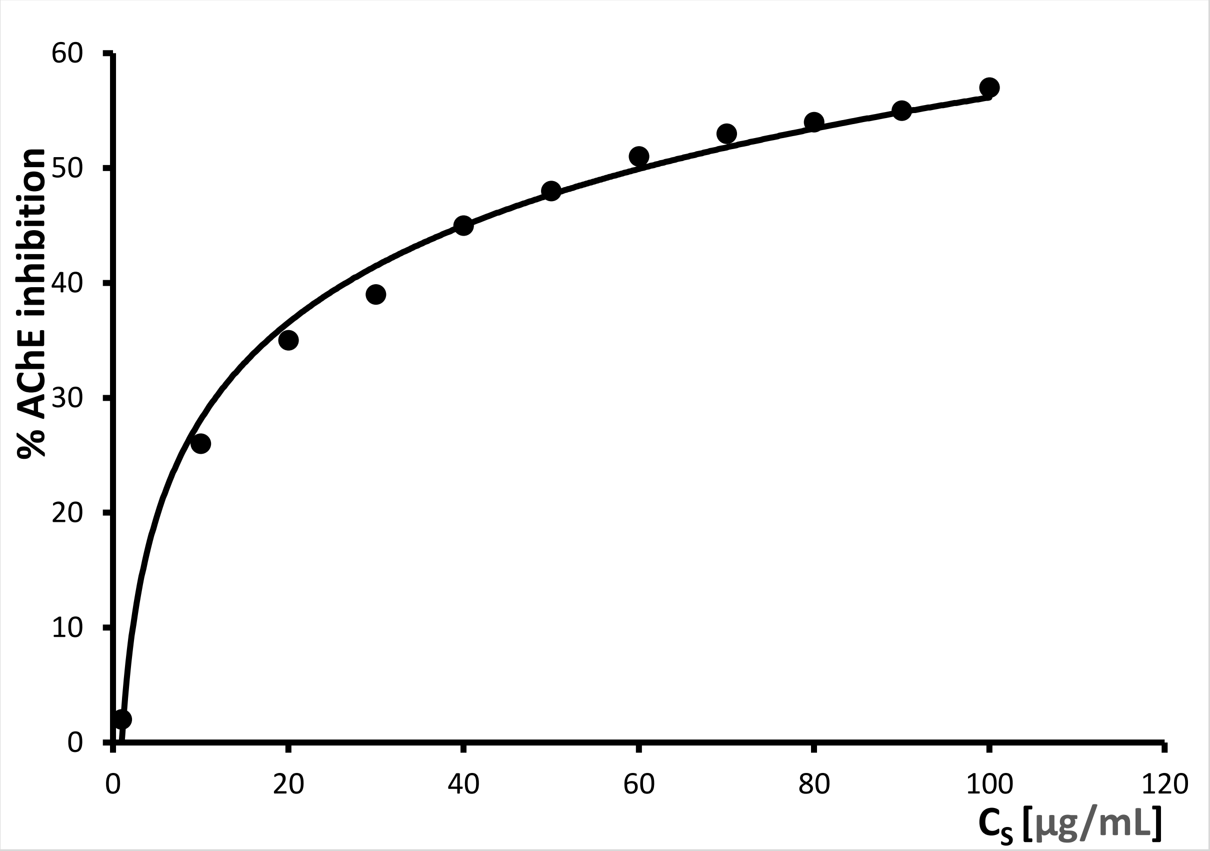

Figure S3B. The dose of extract obtained from *Macleaya cordata* root collected in May response curve of butyrylcholinesterase inhibitory activity.

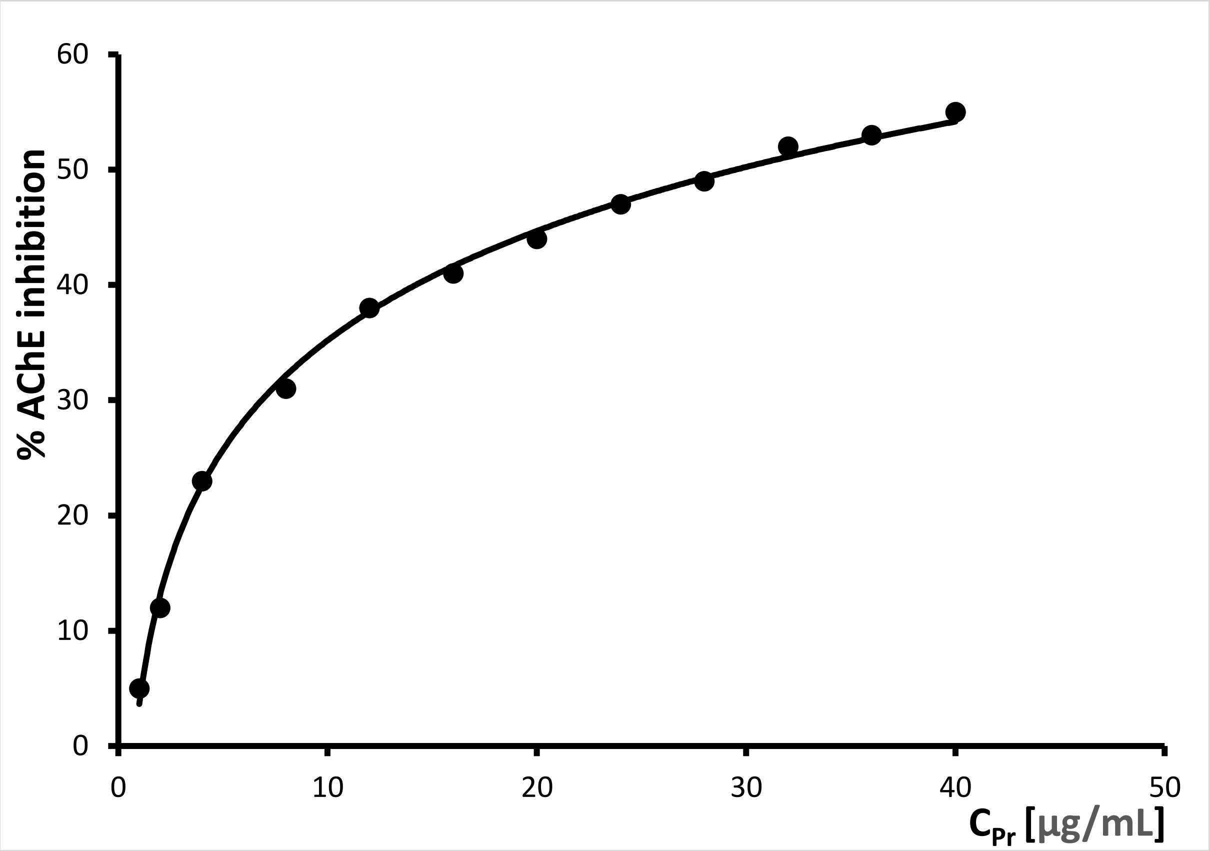

Figure S3C. The dose of extract obtained from *Macleaya cordata* aerial part collected in July response curve of butyrylcholinesterase inhibitory activity.

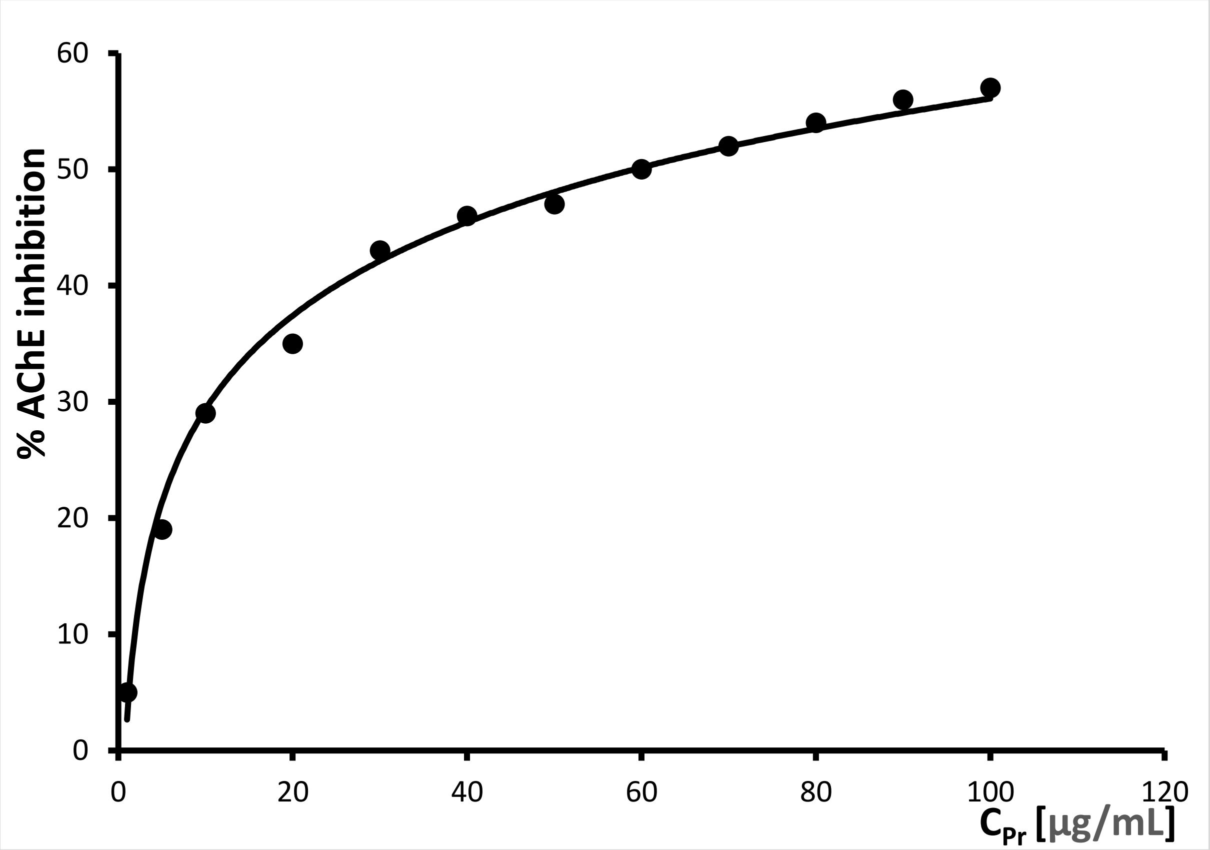

Figure S3D. The dose of extract obtained from *Macleaya cordata* root collected in July response curve of butyrylcholinesterase inhibitory activity.

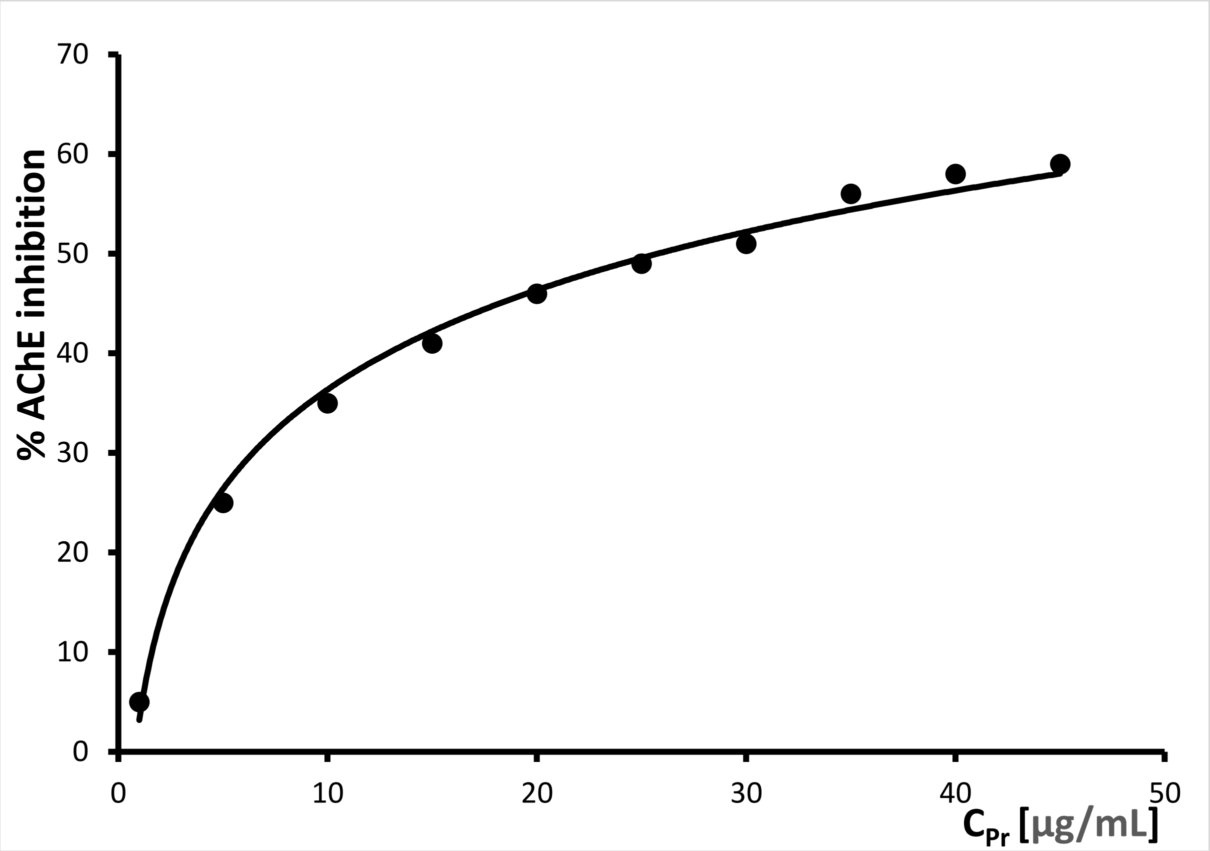

Figure S3E. The dose of extract obtained from *Macleaya cordata* aerial part collected in September response curve of butyrylcholinesterase inhibitory activity.

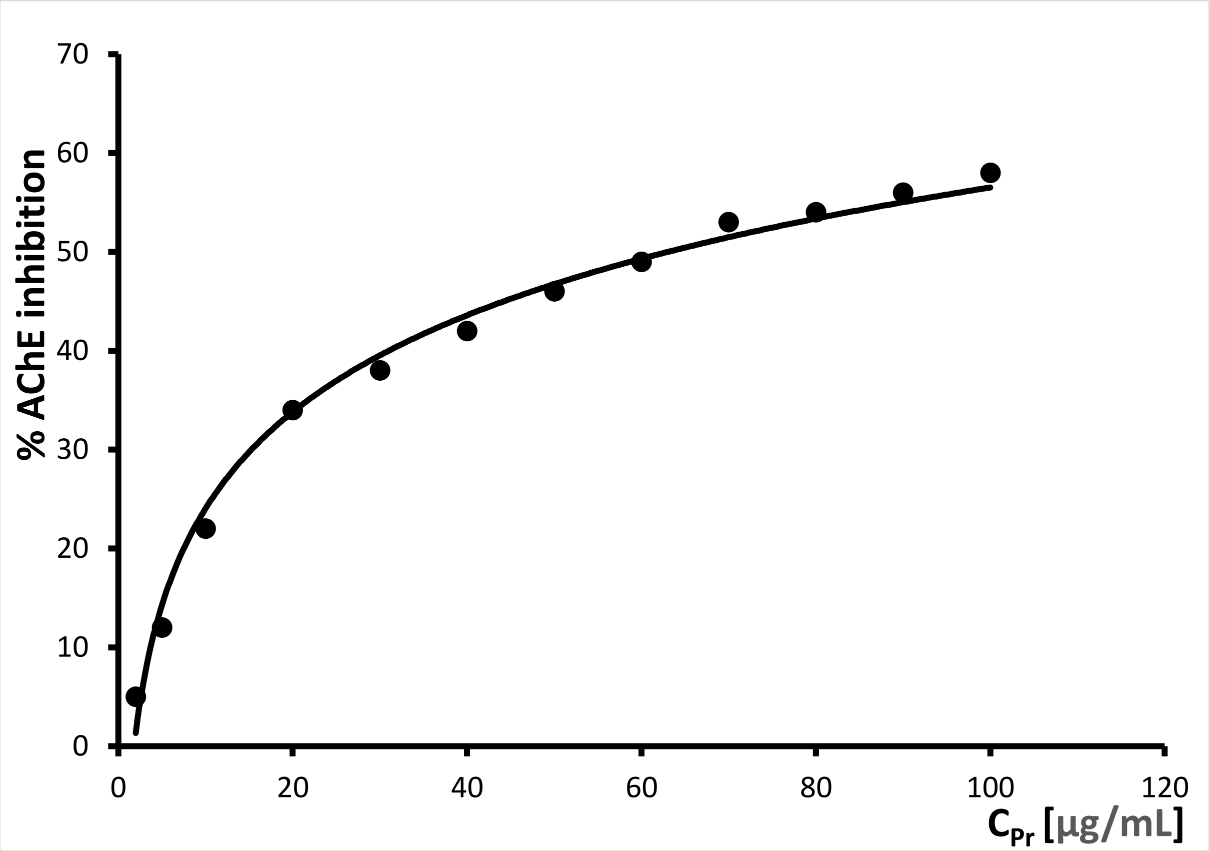

Figure S3F. The dose of extract obtained from *Macleaya cordata* root collected in September response curve of butyrylcholinesterase inhibitory activity.
